# Limited Predictability of Amino Acid Substitutions in Seasonal Influenza Viruses

**DOI:** 10.1093/molbev/msab065

**Published:** 2021-03-05

**Authors:** Pierre Barrat-Charlaix, John Huddleston, Trevor Bedford, Richard A. Neher

**Affiliations:** 1 Biozentrum, Universität Basel, Basel, Switzerland; 2 Swiss Institute of Bioinformatics, Basel, Switzerland; 3 Molecular and Cellular Biology Program, University of Washington, Seattle, WA, USA; 4 Vaccine and Infectious Disease Division, Fred Hutchinson Cancer Research Center, Seattle, WA, USA

**Keywords:** evolution, influenza, population genetics

## Abstract

Seasonal influenza viruses repeatedly infect humans in part because they rapidly change their antigenic properties and evade host immune responses, necessitating frequent updates of the vaccine composition. Accurate predictions of strains circulating in the future could therefore improve the vaccine match. Here, we studied the predictability of frequency dynamics and fixation of amino acid substitutions. Current frequency was the strongest predictor of eventual fixation, as expected in neutral evolution. Other properties, such as occurrence in previously characterized epitopes or high Local Branching Index (LBI) had little predictive power. Parallel evolution was found to be moderately predictive of fixation. Although the LBI had little power to predict frequency dynamics, it was still successful at picking strains representative of future populations. The latter is due to a tendency of the LBI to be high for consensus-like sequences that are closer to the future than the average sequence. Simulations of models of adapting populations, in contrast, show clear signals of predictability. This indicates that the evolution of influenza HA and NA, while driven by strong selection pressure to change, is poorly described by common models of directional selection such as traveling fitness waves.

## Introduction

Seasonal influenza A viruses (IAV) infect about 10% of the global population every year, resulting in hundreds of thousands of deaths (Petrova and Russell 2017; World Health Organization 2018). Vaccination is the primary measure to reduce influenza morbidity. However, the surface proteins hemagglutinin (HA) and neuraminidase (NA) continuously accumulate mutations at a high rate, leading to frequent antigenic changes (Shih et al. 2007; Bhatt et al. 2011; Koel et al. 2013; Petrova and Russell 2017). Although a vaccine targeting a particular strain may be efficient for some time, antigenic drift will sooner or later render it obsolete. The World Health Organization (WHO) regularly updates influenza vaccine recommendations to best match the circulating strains. Since developing, manufacturing, and distributing the vaccine takes many months, forecasting the evolution of influenza is of essential interest to public health (Klingen, Reimering, Guzmán, et al. 2018; Morris et al. 2018).

The number of available high-quality HA and NA sequences has increased rapidly over the last 20 years (Bogner et al. 2006; Shu and McCauley 2017) and virus evolution and dynamics can be now be tracked at high temporal and spatial resolution (Rambaut et al. 2008). This wealth of data has given rise to an active field of predicting influenza virus evolution (Klingen, Reimering, Guzmán, et al. 2018; Morris et al. 2018). These models predict the future population of influenza viruses by estimating strain fitness or proxies of fitness. Luksza and Lässig (2014), for example, train a fitness model to capture antigenic drift and protein stability on patterns of epitope and nonepitope mutations. Other approaches by Steinbrück et al. (2014); Neher et al. (2016); Huddleston et al. (2020) predict fitness by using hemagglutination inhibition (HI) data to determine possible antigenic drift of clades in the genealogy of the HA protein. Finally, Neher et al. (2014) use branching patterns of HA phylogenies as a proxy for fitness. These branching patterns are summarized by the Local Branching Index (LBI), which was shown to be a proxy of relative fitness in mathematical models of rapidly adapting populations (Neher et al. 2014).

The underlying assumption of all these methods is that 1) differences in growth rate between strains can be estimated from sequence or antigenic data and 2) that these growth rate differences persist for long enough to be predictive of future success. Specific positions in surface proteins are of particular interest in this context. The surface proteins are under a strong positive selection and change their amino acid sequence much more rapidly than other IAV proteins or than expected under neutral evolution (Bhatt et al. 2011; Strelkowa and Lässig 2012). Epitope positions, that is, positions targeted by human antibodies, are expected to change particularly often since viruses with altered epitopes can evade existing immune responses (Wolf et al. 2006; Shih et al. 2007; Koel et al. 2013). It therefore seems plausible that mutations at these positions have a tendency to increase fitness and a higher probability of fixation (Strelkowa and Lässig 2012). But one has to be careful to account for the fact that these positions are often ascertained post hoc (Shih et al. 2007) and human immune responses are diverse with substantial interindividual variation (Lee et al. 2019).

A mutation with a fitness benefit should have a tendency to fix, whereas detrimental mutations fix less often. Fixation and loss of a mutation is, however, not only determined by its intrinsic fitness benefit, but also by the fitness of the background it arose on and the fitness of competing variants (Strelkowa and Lässig 2012). The degree to which these different components affect influenza evolution has been investigated by Illingworth and Mustonen (2012), who showed that intrinsic selection, background fitness, and later interference have comparable effects on fixation. These interference effects, along with stochastic epidemiological dynamics and transient selection, limit the predictability of evolution. Here, we investigate the frequency trajectories of amino acid mutations and the degree to which fixation or loss are predictable. We quantify how rapidly mutations at different frequencies are lost or fixed and how rapidly they spread through the population. We further investigate whether any properties or statistics are predictive of whether a particular mutation fixes or not. To our surprise, we find that the predictability of these trajectories is very limited: for A/H3N2, the probability that a mutation fixes differs little from its current frequency, as would be expected if fixation happened purely by chance. This observation holds for many different categories of mutations, including mutations at epitope positions. This weak predictability is not attributable solely to clonal interference and genetic linkage, as simulation of models including even strong interference retains clear signatures of predictability. Consistent with these observations, we show that a simple predictor uninformed by fitness, the consensus sequence, performs as well as the LBI, the growth measure based on the genealogy used in (Neher et al. 2014). This suggests that although LBI has predictive power, the reason for its success may not be related to it approximating fitness of strains.

## Results

The main underlying question asked in this work is the following: given a mutation *X* in the genome of influenza that we observe at a frequency *f* in the population at a given date, what can we say about the future of *X*? The trajectory of a mutation will depend on its own effect on fitness, the contribution of the genetic background on the same segment, and the effect of the remaining seven segments. Here, we investigate properties of broad categories of mutations effectively averaging over different genetic backgrounds to isolate the effects intrinsic to novel mutations in this category.

First, we ask whether we can quantitatively predict the frequency of *X* at future times *f*(*t*). In other words, having observed a mutation at frequencies $\left(f_{1},f_{2},\ldots ,f_{n}\right)$ at dates $\left(t_{1},t_{2},\ldots ,t_{n}\right)$, what can we say about its frequency at future dates $\left(t_{n+1},t_{n+2},\ldots \right)$? A simpler, more qualitative question, is to ask whether *X* will fix in the population, will disappear, or whether the site will stay polymorphic.

We use amino-acid sequences of the HA and NA genes of A/H3N2 (since the year 2000) and A/H1N1pdm (since the year 2009) influenza available in GISAID (Shu and McCauley 2017) (see Supplementary Material online for an acknowledgment of all data contributors). This amounts to 44,976 HA and 36,300 NA sequences for A/H3N2 and 45,350 HA and 40,412 NA sequences for A/H1N1, with a minimum of 100 per year. These sequences are binned in nonoverlapping intervals of 1 month. Each single-month time bin and the sequences that it contains represent a (noisy) snapshot of the influenza population at a given date. The number of sequences per time bin varies strongly both with year and according to the season, with earlier time bins containing around ten sequences while more recent bins contain several hundreds (see supplementary figs. S9 and S10, Supplementary Material online, for details).

The central quantities that we derived from this data are “frequency trajectories” of amino acids at each position in the sequences. If an amino acid *X_i_* is found at position *i* at a frequency between 5% and 95% in the population of a given time bin *t*, then the population is considered polymorphic at position *i* and at time *t*. This polymorphism is characterized by the frequency of *X_i_*, $f_{X_{i}}(t)$, and also by frequencies of other amino acids at *i*. The series of values $f_{X_{i}}(t)$ for contiguous time bins constitutes the frequency trajectory of *X_i_*. A trajectory is terminated if the corresponding frequency is measured above 95% (resp. below 5%) for two time bins in a row, in which case amino acid *X_i_* is considered as “fixed” (resp. absent) in the population. Otherwise, the trajectory is considered “active.” Examples of trajectories can be seen in supplementary figure S11, Supplementary Material online. We focus on frequency trajectories that are starting at a zero (low) frequency, that is, f(t=0)=0. These represent new amino acid variants which were absent in the population at the time bin when the trajectory started and are currently rising in the population (see Materials and Methods).

Since genomic segments of influenza do not recombine, concurrent trajectories of mutations on the same segment will be correlated. In the extreme cases where the two mutations always appear in the same strains, their two trajectories will be identical. This “nesting” of frequency trajectories may introduce biases when performing statistical analysis. In supplementary section S3, Supplementary Material online, we investigate the effect of such nested trajectories by clustering them based on the strains that compose them. Although the number of trajectories that contribute to the analysis decreases with more aggressive clustering, it does not significantly change the results presented below.

### Predicting Future Frequencies

**Fig. 1.**
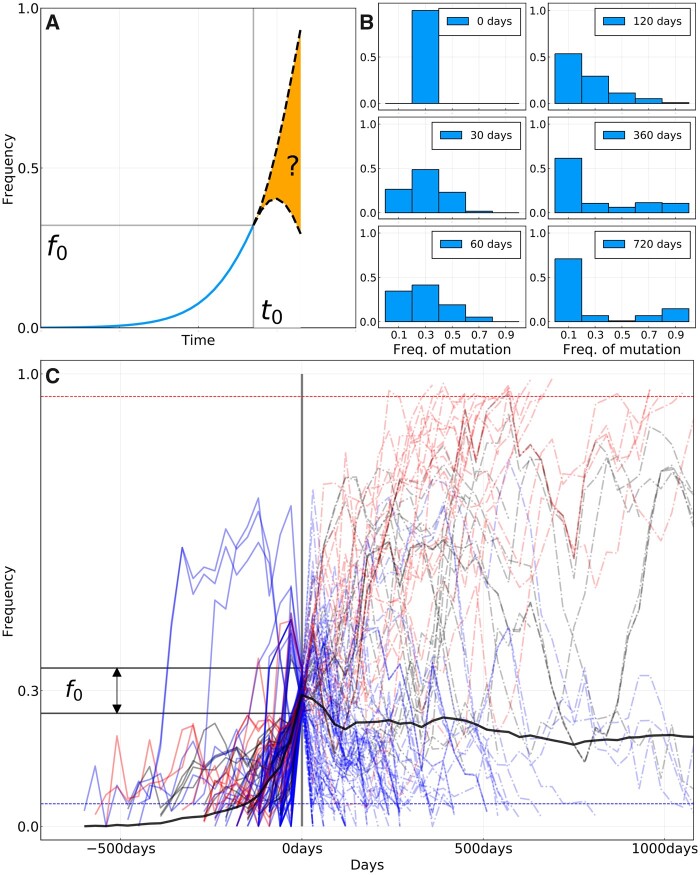
(*A*) Sketch of the idea behind the short-term prediction of frequency trajectories. Given a mutation that we have seen increasing in frequency and that we “catch” at frequency *f*_0_ at time *t*_0_, what can we say about the distribution of future frequencies $P_{\Delta t}(f|f_{0})$? (*B*) Distribution of future frequencies $P_{\Delta t}(f|f_{0})$ for the trajectories shown in (*C*) and for specific values of Δt. (*C*) All frequency trajectories of amino acid mutations in the A/H3N2 HA and NA genes that were absent in the past, are seen around $f_{0}=30\%$ frequency at time $t_{0}=0$, and are based on more than ten sequences at each time point. Red curves represent mutations that will ultimately fix, blue the ones that will be lost, and black the ones for which we do not know the final status. Dashed horizontal lines (blue and red) represent loss and fixation thresholds. The thick black line is the average of all trajectories, counting those that fix (resp. disappear) as being at frequency 1 (resp. 0). Supplementary figure S12, Supplementary Material online, shows equivalent figures for other values of *f*_0_.

Having observed the frequency trajectory *f*(*t*) of a mutation until a given date *t*_0_, how much can we say about the future values of *f* after *t*_0_? We consider the idealized case sketched in figure 1*A*: given the trajectory of a “new” mutation, that is, that started at a frequency of 0, and that we observe at frequency *f*_0_ at time *t*_0_, what is the probability $P_{\Delta t}(f|f_{0})$ of observing it at a value *f* at time $t_{0}+\Delta t$? Such frequency propagators were originally proposed by (Strelkowa and Lässig 2012) to quantify the strength of selection in influenza virus evolution.

To answer this question retrospectively, we use all frequency trajectories extracted from HA and NA sequences that satisfy these conditions for a given *f*_0_. The number of trajectories is limited and the frequency estimates themselves are based on a finite sample and are hence imprecise. Therefore, we consider trajectories in an interval $\left[f_{0}-\delta f,f_{0}+\delta f\right]$ with δf=0.05.

For $f_{0}=0.3$, we found 120 such trajectories in the case of A/H3N2 influenza, represented in figure 1*C*, where time is shifted such that $t_{0}=0$. The same analysis was performed for A/H1N1pdm, with the 89 found trajectories displayed in supplementary figure S13, Supplementary Material online. Some trajectories fall in the frequency bin around *f*_0_ while decreasing, even though they crossed that bin at an earlier time. This is due to the fact that some trajectories “skipped” the interval *f*_0_ in question on their initial rise due to sparse sampling. These trajectories are nevertheless rising in the sense that they start at frequency 0 for t→−∞. Removing them does not change results significantly.

Since rapid sequence evolution of influenza HA and NA mediates immune evasion, one could expect that a significant fraction of new amino acid mutations on rising trajectories in figure 1 are “adaptive.” We could thus expect that most of these trajectories continue to rise after reaching frequency *f*_0_, at least for some time. A fraction of those would then sweep through the population and fix.

To quantify the extent to which this preconception of sweeping adaptive mutations is true, we estimated the probability distribution $P_{\Delta t}(f|f_{0})$ of finding a trajectory at frequency *f* after a time Δt given that it was observed at *f*_0_ at time 0. The results for different Δt are shown in figure 1*B*. Initially, that is, at time $t_{0}=0$, this distribution is by construction peaked around *f*_0_. If a large fraction of the trajectories keep increasing after this time, we should see the “mass” of $P_{\Delta t}(f|f_{0})$ move to the right toward higher frequencies as time progresses.

However, future distributions for Δt>0 do not seem to follow a pattern compatible with selective sweeps. The thick black line in figure 1*C* shows the average frequency of all trajectories. This average makes a sharp turn at *t *=* *0 and is essentially flat for *t *>* *0 in the case of A/H3N2, and slightly increasing for A/H1N1pdm (supplementary fig. S13, Supplementary Material online). Hence, the fact that this average rose for *t *<* *0 gives little information for *t *>* *0, and is due to the conditions by which these trajectories were selected. This shows that sweep-like trajectories rising steadily from frequency 0 to 1 are not common enough to dominate the average trajectory.

Consistent with the average, the frequency distribution of the selected trajectories broadens in time without a significant shift of the mean as time passes. After 60 days, the distribution is rather symmetrical around the initial $f_{0}=0.3$ value, suggesting that the knowledge that the trajectories were rising is lost after 2 months. On a timescale of 60 to 120 days, the only possible prediction is that trajectories are likely to be found in a broad interval around the initial frequency *f*_0_. After 1 year, the distribution becomes almost flat (excluding mutations that have disappeared or fixed), and the initial peak at *f*_0_ is not visible anymore. The only information remaining from the initial frequency is the fraction that fixed or was lost (see below). This behavior is expected in neutral models of evolution (Kimura 1964) but incompatible with a dynamic dominated by sweeps taking over the population.

In contrast, the same analysis for A/H1N1pdm (supplementary fig. S13, Supplementary Material online) reveals a consistent upward trend of novel frequency trajectories. In A/H3N2, however, past dynamics alone is uninformative of future dynamics.

### Prediction of Fixation or Loss

**Fig. 2.**
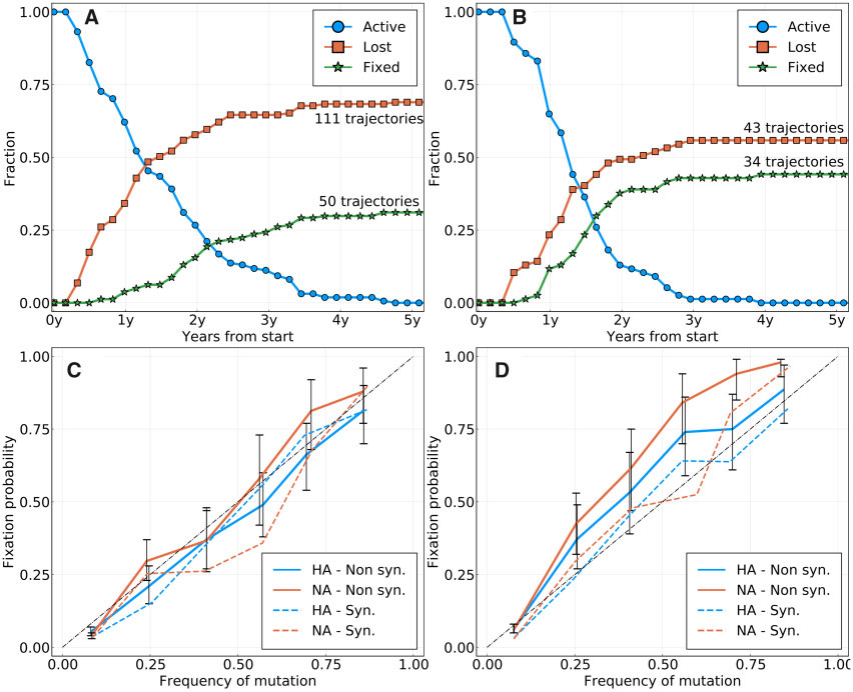
(*A*) Activity of all rising frequency trajectories seen above 25% frequency for A/H3N2 HA and NA. (*B*) Same as (*A*) for A/H1N1pdm. (*C*) Probability of fixation of a mutation (amino acid or synonymous) $P_{\text{fix}}(f)$ as a function of the frequency *f* at which it is measured, for A/H3N2 HA and NA. Only new mutations are considered, that is, mutations that were absent in the past. The diagonal dashed line is the expectation from a neutrally evolving population. Colored dashed lines represent synonymous mutations. Colored solid lines represent amino acid mutations. Error bars represent a 95% confidence interval. (*D*) Same as (*C*) for A/H1N1pdm.

Instead of predicting future frequency, we then considered the probability that a mutation fixes in the population. We first estimate the fraction of frequency trajectories that either fix in the population or are lost, as well as the time it takes for one or the other to happen. Figure 2*A* and *B* shows the fraction of frequency trajectories in HA and NA that either have fixed, were lost or remained active as a function of the time elapsed since they were first seen above 25% frequency for A/H3N2 and A/H1N1pdm, respectively. Most mutations are either lost or become fixed after 2–3 years, with very few trajectories remaining active after 5 years. This time scale of 2–3 years is consistent with the typical coalescence time observed in phylogenetic trees of A/H3N2 influenza (Rambaut et al. 2008; Yan et al. 2019). We also note that the fraction of lost trajectories increases sharply at small times with 40% of mutations observed above 25% frequency being lost within 1 year for A/H3N2, whereas it takes longer to fix a mutation in the whole population.

We then examined the probability of mutations to fix in the population as a function of the frequency at which they are seen. For different values of frequency *f*, we consider all trajectories that started at a null frequency and are seen in the interval [f−7.5%,f+7.5%] at any given time. The fixation probability of a mutation at frequency *f*, $P_{\text{fix}}(f)$, is then estimated by the fraction of those trajectories which terminate at a frequency larger than 95%, that is, our fixation threshold. Figure 2*C* and *D* shows $P_{\text{fix}}(f)$ as a function of *f* for NA and HA.

In the “absence” of selection, we expect $P_{\text{fix}}(f)$ to equal *f*. A mutation or trait appearing at frequency *f* is shared by f·N individuals, and the probability for one of them to become the ancestor of all the future population is f·N/N=f. Thus, the probability of this mutation or trait to fix in the population is equal to its current frequency, a case which we will refer to as the neutral expectation. Figure 2*C* indicates that mutations in the surface proteins of A/H3N2 influenza are in good agreement with the neutral expectation. For A/H1N1pdm, we find that new amino acid mutations fix moderately but consistently more often than their current frequency, whereas synonymous mutations conform with the neutral expectation. In all cases, frequency is a strong predictor of fixation.

**Fig. 3.**
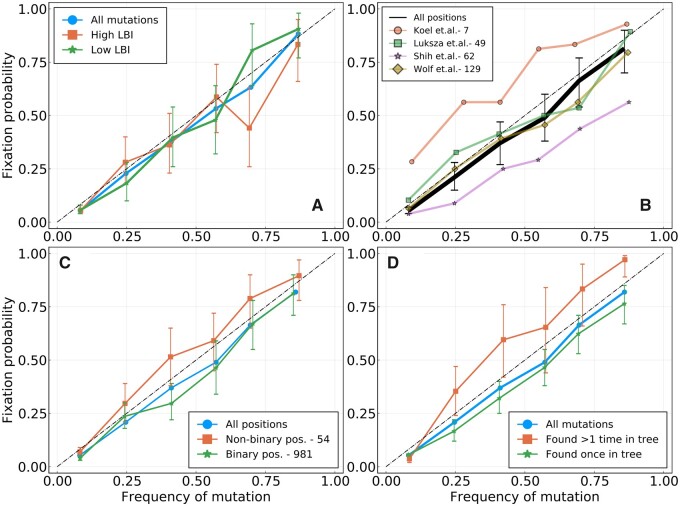
Fixation probability $P_{\text{fix}}(f)$ as a function of frequency, for A/H3N2 influenza. Supplementary figure S15, Supplementary Material online, shows the same analysis for A/H1N1pdm. (*A*) HA mutations with higher or lower LBI values, based on their position with respect to the median LBI value. (*B*) Different lists of epitope positions in the HA protein. The authors and the number of positions are indicated in the legend. (*C*) HA and NA mutations for binary positions, that is, positions for which we never see more than two amino acids in the same time bin. (*D*) HA and NA mutations that appear once or more than once in the tree for a given time bin.

Next, we searched for features of mutations in A/H3N2 that allow prediction of fixation beyond frequency. We first turn to the LBI, a quantity calculated for each node in a phylogenetic tree that indicates how dense the branching of the tree is around that node. LBI has previously been successfully used as a predictor of the future population of influenza (Neher et al. 2014), and was shown to be a proxy for fitness of leaves or ancestral nodes in mathematical models of evolution. Here, we define the LBI of a mutation at date *t* as the average LBI of strains that carry this mutation and that were sampled in the time bin corresponding to *t*. Figure 3*A* shows fixation probability for HA mutations with LBI in the top or bottom half of the distribution. Both groups have indistinguishable probabilities of fixation, suggesting that LBI carries very little information on the probability of fixation of a mutation.

Next, we focused on previously reported antigenic sites in the A/H3N2 HA protein, referred to as “epitope” positions. Mutations at these positions might mediate immune escape and might be expected to be under strong selection and show sweep-like behavior. We used four lists of relevant epitope positions from different sources comprising from 7 to 129 positions in the sequence of the HA1 protein (Wolf et al. 2006; Shih et al. 2007; Koel et al. 2013; Luksza and Lässig 2014). Figure 3*B* shows fixation probability as a function of frequency for the four lists of epitopes. Only mutations at the seven pivotal sites reported in (Koel et al. 2013) have higher chances of fixation than expected by chance. No clear difference is found for the lists by Luksza and Lässig (2014); Wolf et al. (2006), whereas positions from Shih et al. (2007) fix less often. One should also note that many of these positions were determined post hoc and might be enriched for positions that experienced rapid substitutions before the publication of the respective studies.

In supplementary figures S3 and S4, Supplementary Material online, we repeat this analysis for the best predictors from our recent comparison of predictive models for influenza (Huddleston et al. 2020). We consider individual predictors based on LBI, mutational load, variation in frequency, and serological data, as well as two combinations of these quantities. Although many of these models predict future population composition, the only score that was predictive of fixation is mutational load.

In addition to mutational load, we find that parallel evolution is predictive of fixation. In figure 3*C*, we split trajectories into those occurring at binary positions where only two amino acid variants cocirculate and nonbinary positions with more than two variants. Novel variants at nonbinary positions, that is, ones for which competition between three amino acids or more has occurred at least once, have a higher chance of fixation. In figure 3*D*, we separated mutations that appear more than once or only once in the reconstructed tree (see Materials and Methods), and found that the former fix more often. Figure 3*C* and *D* shows that it is possible to gain some information on the chance of fixation of a particular mutation, as was done in figure 3*B*. However, the predictive power remains small, with the “top” curves in figure 3*C* and *D* being very close to the diagonal.

We conducted the same analysis for A/H1N1pdm, with results shown in supplementary figure S15, Supplementary Material online. Results are qualitatively similar to those obtained for A/H3N2, with LBI giving little information and mutations at nonbinary positions having a higher chance of fixation. Panel (*D***)** differs between figure 3 and supplementary figure S15, Supplementary Material online, with convergent evolution giving less information on fixation for A/H1N1pdm. However, the number of recurring mutations for A/H1N1pdm in (***D*)** of supplementary figure S15, Supplementary Material online, is small and confidence intervals large.

Since influenza is seasonal in temperate regions, geographic spread and persistence might be predictive of the success of mutations. We quantify geographic spread of a mutation by the entropy of its frequency distribution across regions (see Materials and Methods) and its persistence by the age of the trajectory by the time it reaches frequency *f*. Supplementary figures S16 and S17, Supplementary Material online, show the fixation probabilities as a function of observed frequency for mutations classified according to these scores. The two scores also allow a quantitatively moderate distinction between mutations: for a given frequency *f*, mutations found in many regions or those that are older (in the sense that they have taken more time to reach frequency *f*) tend to fix more often than geographically localized mutations or more recent ones, but the effect is small. These two scores are in fact correlated, with older trajectories representing mutations that are more geographically spread, as can be seen in supplementary figure S18, Supplementary Material online. However, it is important to note that sampling biases and heterogeneity across time and space (see supplementary figs. S9 and S10, Supplementary Material online) make answering such specific hypothesis challenging.

### Simulations of Models of Adaptation

The results shown in figures 2 and 3 are difficult to reconcile with the idea that seasonal influenza virus evolution is driven by rapid directed positive selection. One possible explanation for the weakly predictable behavior of mutations (beyond their current frequency) might be tight genetic linkage inside each segment and strong competition between different adaptive mutations (Neher and Shraiman 2011; Strelkowa and Lässig 2012). Indeed, Illingworth and Mustonen (2012) have shown that background fitness and interference have substantial effects on frequency trajectories.

To investigate the degree to which interference can explain the observations, we simulate a simple model of influenza evolution using ffpopsim (Zanini and Neher 2012). The model represents a population of binary genomes of length *L *=* *200 evolving in a fitness landscape that changes through time. A similar setting was used in (Strelkowa and Lässig 2012).

First, we use an additive fitness function, with sequence $\left(x_{1}\ldots x_{L}\right)$ having a fitness $\sum_{i}h_{i}x_{i}$. This implies that for a given genome position *i*, the trait *x_i_ *=* *1 is favored if $h_{i}>0$ whereas $x_{i}=-1$ is favored if $h_{i}<0$. All *h_i_*’s have the same magnitude, and only their signs matter. Every Δt generations, we randomly choose a position *i* and flip the sign of *h_i_*, effectively changing the fitness landscape. Individuals in the population now have the opportunity to make an adaptive mutation at site *i* giving them a fitness advantage 2|h|. A “flip” at position *i* of the fitness landscape will decrease fitness of all individuals that carried the adapted variant at position *i* and increases the fitness of those that happened to carry a deleterious variant.

To increase competition between genomes, we designed a second model that includes epistasis. Once again, the baseline fitness of a genome is an additive function, this time with values of *h_i_* that do not change through time. In addition, we added a component that mimics immune selection. Every Δt generations, we introduce “antibodies” that target a specific subsequence of length *l *=* *5, noted $\left(x_{i_{1}}^{ab},\ldots ,x_{i_{l}}^{ab}\right)$. The positions $\left(i_{1}\ldots i_{l}\right)$ are chosen at random, whereas the targeted subsequence is the dominant state at each position. Genomes that include the “exact” subsequence targeted by the antibody suffer a strong fitness penalty. However, a single mutation away from that subsequence removes this penalty completely. This has the effect of triggering a strong competition between adaptive mutations: for a given antibody, *l *=* *5 possible mutations are now adaptive, but combinations of these mutations do not bring any fitness advantage.

Having simulated populations in these two fitness landscapes, we perform the same analysis of frequency trajectories as for the real influenza data. Supplementary figure S20, Supplementary Material online, shows the $P_{\text{fix}}(f)$ as a function of *f* for the two models and for different values of the inverse rate of change Δt of the fitness landscape. Increasing interference reduces $P_{\text{fix}}(f)$ but even for very strong interference $P_{\text{fix}}(f)$ remains significantly larger than *f*.

For a fairly stable simple additive fitness landscape (Δt=1000) rising mutations almost always fix, with $P_{\text{fix}}(f)≃1$ for any *f* larger than a few percent. This is corroborated by visual inspection of the trajectories, which shows that evolution in this regime is driven by regular selective sweeps that take a typical time of ∼400 generations. Smaller Δt or strong epistatic competition reduce $P_{\text{fix}}(f)$. However, it takes an extremely fast-changing fitness landscape to push *P*_fix_ close to the diagonal: with Δt=10, that is about 40 changes to the fitness landscape in the time it would take a selective sweep to go from 0% to fixation, $P_{\text{fix}}(f)$ differs from *f* in a way that is comparable to what is observed in A/H1N1pdm influenza.

Next, in the case of a fast-changing fitness landscape (Δt=10), we cluster trajectories of simulated populations based on the intrinsic fitness effect of corresponding mutations. Since competition between adaptive mutations and genetic linkage is very strong in this regime, we do not expect the intrinsic fitness effect to be fully predictive of fixation. However, supplementary figure S21, Supplementary Material online, shows that mutations with an above median fitness effect have a significantly larger chance to fix than those with a below median fitness effect. This strongly contrasts with figure 3, and confirms the intuition that intrinsic fitness effect can be partly predictive of fixation even in a regime of strong clonal interference.

These models are not meant to be accurate models of influenza viruses evolution. But supplementary figure S20, Supplementary Material online, does show that the patterns observed in influenza virus evolution are only reproduced by models of adapting populations when pushing clonal competition to extreme values. We conclude that the quasi-neutral pattern in figure 2 may not be a straightforward manifestation of genetic linkage and clonal interference, but that some more intricate interplay of epidemiology, seasonality, human immunity, and chance gives rise to the weakly predictable yet strongly selected evolutionary dynamics of IAVs.

### Why Do Predictions Work?

The statistics of frequency trajectories seem to be in conflict with the notion that influenza evolution is predictable. Likewise, the LBI, a quantity that correlates with fitness in mathematical models and is used to predict future influenza populations (Neher et al. 2014), does not seem to contain any information on whether a specific mutation is going to fix or not, see figure 3. To resolve this conundrum, we first note that the criterion by which predictive power for influenza was measured in (Neher et al. 2014) was the distance between the strain with the highest LBI and the future population, not the ability of the LBI to predict dynamics. The distance was compared with the average distance between the present and future population, as well as the post hoc optimal representative and the future.

To quantify the ability of the LBI and other measures to pick good representatives of the future, we construct a large tree of HA sequences with 100 sequences in nonoverlapping time bins of 4 months from years 2003 to 2019 (a total of 4,402 as some 4-month intervals contain less than 100 sequences). Each time bin is considered as a snapshot of the A/H3N2 influenza population and we will refer to sequences in time bin *t* as the population of the “present.” From this present population, we predict “future” populations in time bin t+Δt, using only sequences in time bin *t* and before.

**Fig. 4.**
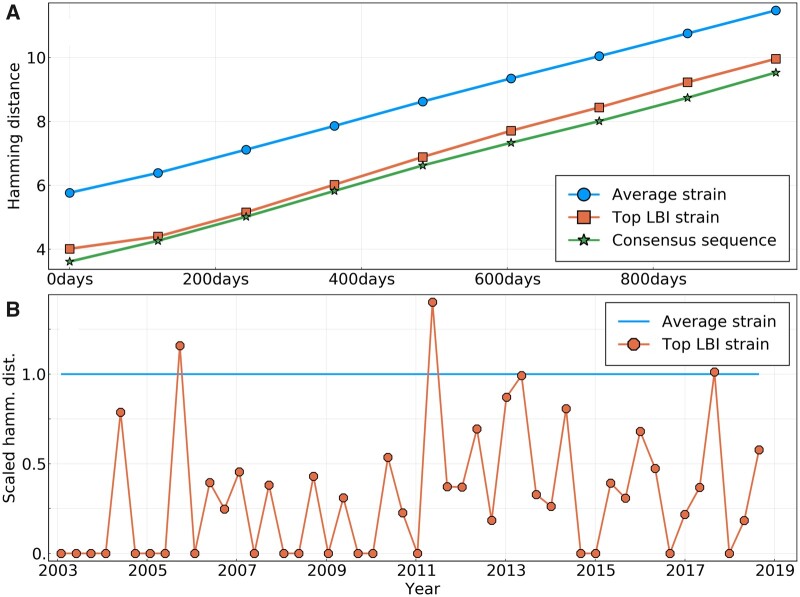
(*A*) Average amino acid Hamming distance of the sequences of different predictors to HA sequences of future influenza populations, themselves averaged over all “present” populations from years 2003 to 2019. Predictors are: a randomly picked sequence in the present population; the sequence of the strain with the highest LBI in the present population; the consensus sequence of the present population. (*B*) Scaled Hamming distance between the sequence of the top LBI strain and the consensus sequence for populations at different dates. The scaling is such that for each date, the Hamming distance between a strain from the population and the consensus is on an average 1. The strain with the highest LBI is almost always closer to the consensus sequence than the average strain.

To assess the ability of the LBI to pick a close representative of the future, we compute the LBI of each node of one time bin in the tree using only the leaves that belong to that time bin. The top panel in figure 4 shows the hamming distance of the strain with the highest LBI to future populations at different Δt along with the same distance for a randomly chosen strain. The figure shows the distance averaged over all possible values of *t* for Δt between 0 and 32 months, giving us an average efficiency of a predictor over 16 years of influenza evolution.

The strain with the highest LBI is consistently closer to the future than the average strain by about one to two amino acids, whereas the overall distance increases linearly due to the continuous evolution of the population. We hence reproduce previous results showing that the LBI picks closer than average representatives (Neher et al. 2014). To investigate whether this apparent success is due to the ability of the LBI to predict fitness or not, we explored a different predictor: the amino acid consensus sequence of the present population (see Materials and Methods for a definition of the consensus sequence). The choice is motivated by the fact that it can be shown to be the best possible long-term predictor for a neutrally evolving population in terms of Hamming distance (see supplementary section S1, Supplementary Material online). Figure 4 shows that the consensus sequence is in fact an equally good or even slightly better representative of the future than the sequence with highest LBI (note that the consensus sequence does “not” necessarily exist in the population).

This near equivalence of the consensus and the strain with the highest LBI can be explained as follows: The LBI tends to be high for nodes in a tree that are close to the root of a dense and large clade. A typical sample of influenza HA sequences fall into a small number of recognizable clades, and the strains with maximal LBI will often be close to the root of the largest of those clades. This root of the largest clade will often be close to the consensus of the whole population, explaining the similar distance patterns. To test that hypothesis, we measure the hamming distance from the sequence of the top LBI strain to the consensus sequence for populations of all time bins. Figure 4*B* shows these distances, scaled with respect to an average strain (details in caption). It clearly shows that the top-LBI strain and the consensus sequence are indeed quite similar: out of 48 time bins, only twice is the sequence of the top-LBI strain farther away from the consensus than the average sequence is. Moreover, the sequence of the top-LBI strain “exactly” matches the consensus in 19 cases.

## Discussion

Predicting the trajectory of a mutation requires 1) significant fitness differences between genomes carrying different variants at the site and 2) a selection pressure that changes slowly over time. Under such conditions, it is expected that frequency trajectories will show a persistent behavior which would make them predictable for some time. However, we could find little evidence for such persistent behavior in the past 19 years of A/H3N2 evolution. This leads us to conclude that 1) A/H3N2 influenza virus evolution is qualitatively different from models of rapidly adapting populations (despite clear evidence for frequent positive selection), and 2) many methods to predict influenza evolution are unable to predict future frequency dynamics even though they work well for predicting the composition of the future population. This is illustrated by the case of LBI, which works primarily because it picks strains that represent the future well and not because it is predictive of future dynamics. A/H1N1pdm, in contrast, shows stronger signatures of predictability.

The primary focus in this work was the investigation of frequency trajectories of new amino acid mutations. In the short term, we found that on an average the direction of trajectory does not persist for longer than a few months. Indeed, the average trajectory in figure 1 takes a sharp turn when going from *t *<* *0 to *t *>* *0, instead of showing “inertia.” This suggests that selective sweeps are not representative of typical trajectories (Illingworth and Mustonen 2012).

On a longer timescale, we investigated the probability that a novel mutation observed at frequency *f* fixes. In neutral models of evolution, this probability equals *f*, whereas it should be higher or lower than *f* for mutations with a beneficial or deleterious effect on fitness, respectively. However, in the case of influenza, this probability differs little from *f*, making current frequency the best predictor for fixation. In figure 3 and supplementary figure S3, Supplementary Material online, we split trajectories into groups for which we expected *P*_fix_ to deviate from *f*. Many of these splits, such as high/low LBI or epitope/nonepitope positions, did not result in an increased predictability, whereas others gave limited information on fixation.

Methods for predicting the future evolution of influenza either construct explicit fitness models (Luksza and Lässig 2014; Huddleston et al. 2020), use historical patterns of evolution (Bush et al. 1999; Luksza and Lässig 2014), phenotypic assays (Steinbrück and McHardy 2012; Neher et al. 2016), or dynamic or phylogenetic patterns (Neher et al. 2014; Klingen, Reimering, Loers, et al. 2018). The goal of these methods is to pick strains that are good representatives of future populations and could serve as vaccine candidates (Morris et al. 2018).

The low power to predict frequency dynamics or fixation naturally triggers the question why the above methods have been found to work. Picking representatives of the future and predicting frequency dynamics are distinct objectives and success at the former (as compared with random picks) is not necessarily inconsistent with a lack of predictable dynamics. In fact, Huddleston et al. (2020) reports that the rate at which the frequency of a strain changes is often a poor predictor—consistent with our observations here. But despite the fact that future frequencies are not predicted by the LBI, the strain with the highest LBI in the population is a better predictor of the future population than a randomly picked one. Although the LBI was shown to be a correlate of relative fitness and be predictive of fixation in mathematical models of evolution (Neher et al. 2014), it does not seem to be predictive of influenza evolution because it measures fitness from genealogical structure. Instead, we believe it picks closer than average strains simply because it has the tendency to be maximal at the base of large and dense clades. These basal genotypes are closer to the future populations than the current tips of the tree and hence a better predictor on an average. The consensus sequence of all present strains performs slightly but consistently better than picking the strain with the highest LBI. The consensus sequence is the best possible predictor for a neutrally evolving population, and does not attempt to model fitness in any way.

At the same time, influenza virus surface proteins show strong signatures of selection (Bhatt et al. 2011; Strelkowa and Lässig 2012). Their phylogenies show clear deviations from those expected from the neutral Kingman coalescent, similar to those expected under Bolthausen-Sznitman coalescent (BSC) processes that are generated by traveling wave models of rapid evolution (Desai et al. 2013; Neher and Hallatschek 2013). The correspondence between the BSC and traveling wave models comes from transient exponential amplification of fit strains before these fitness differences are wiped out by further mutation. This exponential amplification generates long-tailed effective offspring distributions which in turn can lead to genealogies described by the BSC (Schweinsberg 2003; Neher and Hallatschek 2013). Many processes other than selection, including seasonality and spatio-temporal heterogeneity, can generate effective long-tailed offspring distributions even in absence of bona fide fitness differences, which might explain ladder-like non-Kingman phylogenetic trees.

A recent study proposed that influenza virus evolution is primarily limited by an asynchrony between population level selection and generation of new variants within infected hosts (Morris et al. 2020). Along these lines, it is possible that the A/H3N2 population readily responds once population level selection is high enough by giving rise to essentially equivalent variants. Furthermore, selection might cause the rapid rise of a novel variant to macroscopic frequencies (observable in a global sample) but its benefit rapidly “expires” because competing variants catch up and/or it mediates immune escape only to a small fraction of the population (Lee et al. 2019). Immunity to A/H1N1pdm might be less variable among humans than to A/H3N2 since A/H1N1pdm has been circulating for only 11 years. Different age-groups therefore got exposed to more similar A/H1N1pdm variants than their initial exposure to A/H3N2. These considerations might explain the disconnect between models of rapid adaptation and the frequency dynamics observed in influenza virus populations.

## Materials and Methods

### Data and Code Availability

The sequences used are obtained from the GISAID database (Shu and McCauley 2017). Acknowledgment tables with strain names, accession numbers, and originating and submitting labs are given as two supplementary files. Outlier strains listed at https://github.com/PierreBarrat/FluPredictibility/tree/master/src/config were removed.

The code used to generate the figures presented here is available at https://github.com/PierreBarrat/FluPredictibility.

### Frequency Trajectories

For a set of sequences in a given time bin, we compute frequencies of amino acids at each position by simple counting. We make the choice of not applying any smoothing method in an attempt to be as close to the data and “model-free” as possible. This is especially important for the short-term prediction of frequency trajectories, as estimations of the “persistence time” of a trajectory might be biased by a smoothing method.

We compute frequency trajectories based on the frequencies of amino acids. A trajectory begins at time *t* if an amino acid is seen under the lower frequency threshold of 5% (resp. above the higher threshold of 95%) for the two time bins preceding *t*, and above this lower threshold (resp. below the higher threshold) for time bin *t*. It ends in the reciprocal situation, that is when the frequency is measured below the lower threshold (resp. above the higher threshold) for two time bins in a row.

In order to avoid estimates of frequencies that are too noisy, we only keep trajectories that are based on a population of at least ten sequences for “each” time bin. As said in the Results section, we also restrict the analysis to trajectories that begin at a 0 frequency, in part to avoid double counting. We find a total of 460 such trajectories. However, only 106 reach a frequency of 20%, on which figure 2 is based for instance.

Note that the fact that we use samples of relatively small sizes—at least for some time bins—can lead to biases in the estimation of frequencies. We show in Supplementary Material online that these biases are generally small and do not induce any qualitative changes to results presented here.

### Local Branching Index

LBI was introduced in (Neher et al. 2014) as an approximation of fitness in populations evolving under persistent selective pressure. The LBI depends only on a phylogenetic tree. It relies on the intuition that the tree below high-fitness individuals will show dense branching events, whereas absence of branching is a sign of low-fitness individuals. Quantitatively, the LBI $\lambda_{i}(\tau )$ of a node *i* is the integral of all of the tree’s branch length around *i*, with an exponentially decreasing weight $e^{-t/\tau }$ with *t* being the branch length. When considering a time binned population, the LBI is computed once for each time bin by considering only the leaves of the tree that belong to the time bin. This means that only branches that ultimately lead to a leaf that belongs to the time bin are considered in the integration.

The time scale *τ* parameterizes the distance over which the tree is informative of the fitness of a particular node. Here, we use a value of *τ* equal to a tenth of $T_{C}≃6$ years, the coalescence time for influenza A/H3N2 strains, converted to units of tree branch length through the average nucleotide substitution rate ($≃4\times 10^{-3}$ substitutions per site per year for HA). We have observed that given our method to predict the future from present populations corresponding to time bins of 4 months, changing the value of *τ* has little effect on the pick of the top LBI strain. By retrospectively optimizing its value, it is possible to reduce the average distance to the population 2 years ahead by ∼0.25 amino acids on an average, making the LBI method almost as good as the consensus on figure 4.

### Measuring the Geographical Spread of a Mutation

For a mutation *X*, we define its regional distribution using the numbers $n_{r}(X)$ that represent the number of sequences sampled in region *r* that carry *X*. Regional weights are then defined as:

$$w_{r}(X)=\frac{n_{r}(X)}{\sum_{r}n_{r}(X)}.$$

We can then measure the geographical spread *G*(*X*) of *X* by using the Shannon entropy of the probability distribution $w_{r}(X)$:

$$G(X)=\sum_{r}w_{r}(X)\;\text{log}(w_{r}(X)).$$

*G*(*X*) is a positive quantity that is larger when *X* is equally present in many regions, and equal to zero when *X* is concentrated in only one region.

Region used are the ones defined in Nextstrain/flu (Hadfield et al. 2018). Those are North America, South America, Europe, China, Oceania, Southeast Asia, Japan & Korea, South Asia, West Asia, and Africa.

### Assigning a Fitness to Trajectories

#### Consensus Sequence

Given a set of *N* sequences $\left(\sigma^{1},\ldots ,\sigma^{N}\right)$ based on an alphabet A (e.g., A has 20 elements for amino acids, four for nucleotides), we can define a “profile” distribution $p_{i}(a)$ by the following expression:

$$p_{i}(a)=\sum_{n=1}^{N}\delta_{\sigma_{i}^{n},a}$$

where *i* is a position in the sequence, $\sigma_{i}^{n}$ the character appearing at position *i* in sequence *σ^n^*, *a* a character of the alphabet and *δ* the Kronecker delta. The profile $p_{i}(a)$ simply represents the fraction of sequences which have character *a* at position *i*.

We then simply define the consensus sequence *σ*^cons^ such that:

$$\sigma_{i}^{\text{cons}}={\text{argmax}}_{a}\;p_{i}(a).$$

In other words, the consensus sequence is the one that has the dominant character of the initial set of sequences at each position.

#### Earth Mover’s Distance

In order to measure the distance of several predictor sequences to the future population, we rely on the Earth Mover’s Distance (EMD), a metric commonly applied in machine learning to compare collections of pixels or words (Rubner et al. 1998; Kusner et al. 2015). Here, we apply it to compute the distance between the sequences of two populations, noted as $X=\left\{\left(x^{n},p^{n}\right)\right\}$ and $Y=\left\{\left(y^{m},q^{m}\right)\right\}$ with n∈{1…N} and m∈{1…M}. In this notation, *x^n^* and *y^m^* are sequences, and *p^n^* and *q^m^* are the frequencies at which these sequences are found in their respective populations. For convenience, we also define $d_{mn}=H(x^{n},y^{m})$ as the Hamming distance between pairs of sequences in the two populations.

We now introduce the following functional:

$$F(w)=\sum_{n,m}d_{nm}w_{nm},$$

with $w=\{w_{nm}\}$ being a matrix of positive weights. The EMD between the two populations X and Y is now defined as the minimum value of function *F* under the conditions:

$$\sum_{n=1}^{N}w_{nm}=q^{m},\;\;\sum_{m=1}^{M}w_{nm}=p^{n},\;\text{and}\;\;w_{nm}\geq 0$$

Intuitively, the weight *w_nm_* tells us how much of sequence *x^n^* is “moved” to sequence *y^m^*. The functional *F* sums all of these moves and attributes them a cost equal to the Hamming distance *d_nm_*. The conditions on weights in **w** ensure that all the weight *p^n^* of *x^n^* is “moved” to elements in Y and vice versa.

The minimization is easily performed by standard linear optimization libraries. Here, we use the Julia library JuMP (Dunning et al. 2017).

## Supplementary Material

Supplementary data are available at *Molecular Biology and Evolution* online.

## Supplementary Material

msab065_Supplementary_Data
