# Supplementary material for: Limited Predictability of Amino Acid Substitutions in Seasonal Influenza Viruses
Source: Mol Biol Evol. 2021 Mar 5;38(7):2767–77. doi: 10.1093/molbev/msab065 (PMC8233509; doi:10.1093/molbev/msab065)
Supplement: msab065_Supplementary_Data [file msab065_supplementary_data.zip › SI.pdf]

SUPPLEMENTARY MATERIAL

1. Consensus sequence as a predictor for neutrally evolving populations

We consider the case of a neutrally evolving and structure-less population, such as the one in the Wright-Fisher model of evolution (Sigwart, 2005). At an initial time $t = 0$, the population consists of N individuals with genomes $(\sigma^1 \ldots \sigma^N)$ of length L (not necessarily distinct).
We make two hypotheses about this population. We first suppose that *no* mutations occur during the evolution of this population. This may seem surprising and is of course not true in the case of influenza. This assumption is however in line with the fact that the object of this work is to predict the outcome of *already existing* mutations in the influenza population. The prediction of mutations that we have not yet seen is not in its scope. Thus, assuming that no new mutations take place can be seen as a simple way to model the fact that we have no information about such events. The second assumption is that the population evolves in a completely neutral way, meaning that the average number of descendants of each genome σ^n is the same. Let us now consider the population after it has evolved for a long time $t \gg T$ where T is the typical coalescence time (for the Wright-Fisher model, $T = 2N$). At this point, all individuals in the future population will descend from a unique individual n_0 in the $t = 0$ population. Our two hypotheses now allow us to make two statements. First, since no new mutations are allowed, the population at $t \gg T$ will be clonal, with all individuals having genome σ^{n_0}. Second, since the evolution is neutral and does not favor any genome in particular, the probability that σ^{n_0} is equal to a given genome σ is $1/N$. In other words, the probability that a genome at $t = 0$ ultimately becomes the ancestor of all the future population is equal to its frequency in the $t = 0$ population.

We now try to find the genome σ that best predicts the future population on the long run, that is for $t \gg T$. Here, we take best to mean that the predictor minimizes $H(\sigma, \sigma^{n_0})$ where H is the Hamming distance defined by

$$H(\sigma^a, \sigma^b) = \sum_{i=1}^{L} (1 - \delta_{\sigma_i^a, \sigma_i^b}), \tag{1}$$

with σ_i being the character appearing at position i of genome σ and δ the Kronecker delta. Since we do not know n_0, we have to average over all its possible values. σ must thus minimize the following quantity:

$$\begin{aligned} \langle H(\sigma, \sigma^{n_0}) \rangle_{n_0} &= \sum_{n=1}^{N} H(\sigma, \sigma^n) \\ &= \sum_{i=1}^{L} \sum_{n=1}^{N} (1 - \delta_{\sigma_i, \sigma_i^n}) \end{aligned} \tag{2}$$

by using the definition of the Hamming distance. We now assume that characters at each positions of the genomes can be indexed by an integer a running from 1 to q. For instance, if these were amino acid sequences, we could index the 20 amino acids by a running from 1 to $q = 20$. We rewrite the Kronecker delta in the previous expression using this indexation:

$$\delta_{\sigma_i, \sigma_i^n} = \sum_{a=1}^{q} \delta_{\sigma_i, a} \delta_{\sigma_i^n, a}.$$

We also introduce the *profile* frequencies $p_i(a)$ of the population at time $t = 0$:

$$p_i(a) = \sum_{n=1}^{N} \delta_{\sigma_i^n, a}. \tag{3}$$

$p_i(a)$ represents the frequency at which character a appears at position i in genomes of the initial population.

Equation 2 now becomes

$$\begin{aligned}\langle H(\sigma, \sigma^{n_0}) \rangle_{n_0} &= \sum_{i=1}^{L} \sum_{n=1}^{N} \left(1 - \sum_{a=1}^{q} \delta_{\sigma_i, a} \delta_{\sigma_i^n, a} \right) \\ &= \sum_{i=1}^{N} \left(1 - \sum_{a=1}^{q} \delta_{\sigma_i, a} p_i(a) \right) \\ &= \sum_{i=1}^{L} (1 - p_i(\sigma_i))\end{aligned} \tag{4}$$

This means that the genome $\sigma = (\sigma_1 \dots \sigma_L)$ which best predicts the future population according to our definition is the one that minimizes the quantity $(1 - p_i(\sigma_i))$ for all positions i. This obviously implies that each σ_i must be chosen as to maximize $p_i(a)$, that is σ_i must be the character that appears the most frequently at position i. Thus, σ must be the *consensus* sequence of the initial population.

2. Predictor based on the local LBI maxima

In figure S19, we use several sequences as a predictor of the future population. Distance between two sets of sequences, *i.e.* the predictor sequences and the ones of the future population, is defined as the Earth Mover's Distance (EMD). Here, we show that for a population evolving under the same hypotheses as in section .1, the best *multiple* sequence long term predictor is again the consensus sequence with weight 1.
Let the predictor be a set of weighted sequences $\{(s^\alpha, q_\alpha)\}$. We again use the fact that in the long term, a unique sequence σ^{n_0} from the present will be the ancestor of the entire population. We want to compute the EMD from the predictor to σ^{n_0}, that is the EMD between the sets $\mathcal{X} = \{(s^\alpha, q_\alpha)\}$ and $\mathcal{Y} = \{\sigma^{n_0}, 1\}$. Applying the definition of the Methods section, it follows that the weights $\mathbf{w}$ are in this case equal to the q_αs. By averaging over all values of n_0, we now obtain

$$\langle \, \mathrm{EMD} \left(\{ (s^\alpha, q_\alpha) \} \right) \rangle_{n_0} = \sum_{n=1}^{N} \sum_{\alpha} H(s^\alpha, \sigma^n) \cdot q_\alpha.$$

By the same calculation procedure as in the previous section, this expression simplifies to

$$\langle \, \mathrm{EMD} \left(\{ (s^\alpha, q_\alpha) \} \right) \rangle_{n_0} = \sum_{i=1}^{L} \left(1 - \sum_{a=1}^{q} p_i(a) q_i(a) \right),$$

where the profile of the present population $p_i(a)$ has already been defined, and $q_i(a)$ stands for the profile of the predictor, that is

$$q_i(a) = \sum_{\alpha} \delta_{s_i^\alpha, a} q_\alpha.$$

To minimize this distance, we find a profile $q_i(a)$ that maximizes the quantity $\sum_\alpha \delta_{s_i^\alpha, a} q_\alpha$ for each position i. It is clear that this is done by assigning a value $q_i(a) = 1$ if a maximizes $p_i(a)$, and $q_i(a) = 0$ otherwise. Thus, the profile of the predictor must be that of the consensus sequence, which is only possible if the predictor becomes $\{\sigma^{cons}, 1\}$.

3. Correcting for nested trajectories

The analysis of the main text computes probabilities of fixation assuming that all trajectories are independent. However, it is well-known that mutations are nested: they appear on backgrounds that already carry other mutations. Since mutations appearing on the same genomes will jointly fix or disappear, many frequency trajectories are not independent but correlated. In order to compensate for potential biases due to this effect, we attempted to cluster trajectories based on similarity in their strain composition. Our aim is that two trajectories corresponding to mutations appearing mostly on the same genomes will be grouped in the same cluster. We then conduct the same analysis as in the main text on a set of *effectively independent* trajectories constructed by taking one trajectory from each cluster.

In order to perform clustering, we define a distance between trajectories. A frequency trajectory of a mutation X is characterized by a series of frequency values $f(t)$ at time point $t \in T$. We define $S(t)$ as the strains that carry mutation X at date t. With this notation, $f(t)$ is the ratio of the number of elements in $S(t)$ to the total number of strains at date t. Let us now consider two frequency trajectories X_1 and X_2.
We define the distance $d(X_1, X_2)$ between these two trajectories based on the average similarity of the strains S_1 and S_2 that compose them:

$$d(X_1, X_2) = 1 - \frac{1}{|T_1 \cap T_2|} \sum_{t \in T_1 \cap T_2} \frac{|S_1(t) \cap S_2(t)|}{|S_1(t) \cup S_2(t)|},$$

where $T_1 \cap T_2$ is the time interval where both trajectories are active, and $|\cdot|$ denotes the number of elements of a set. The quantity summed corresponds to the Jaccard index between strains composing X_1 and X_2 at a given date. It is 1 if the two trajectories share exactly the same strains for this date, and 0 if they share no strain at all. This leads to the two following properties of d:

- if $d(X_1, X_2) = 0$, then X_1 and X_2 represent the same frequency trajectory. The mutations x_1 and x_2 that they correpond to always appear on the same strains and are totally linked.
- if $d(X_1, X_2) = 1$, then X_1 and X_2 can be considered completely independent. This can be the case if the two trajectories do not occur at the same dates, *i.e.* $|T_1 \cap T_2| = 0$, or if their respective mutations are never present on the same genomes.

we attempt to reduce the potential statistical bias due to the nesting of trajectories by grouping them based on the above defined distance. Given a set of trajectories $\{X\}$, we perform a decomposition of $\{X\}$ into disjoint clusters $C_1(d^\star) \cup \ldots \cup C_n(d^\star) = \{X\}$ where $d^\star$ is an arbitrary threshold distance. Clusters are built in such a way that given two trajectories X_i and X_j

$$d(X_i, X_j) \leq d^\star \Rightarrow \exists k : X_i, X_j \in C_k$$

and

$$X_i \in C_k \Rightarrow \exists j \in C_k : d(X_i, X_j) \leq d^\star.$$

These condition imply that the clusters formed are the minimal ones that guarantee that any two trajectories closer than the threshold distance $d^\star$ belong to the same cluster. The number of clusters n depends on the chosen value for $d^\star$.

We compute clusters for different values of $d^\star$ for the case of the HA gene in A/H3N2. The top panel of figure S1 shows the number cluster n as a function of $d^\star$. In the $d^\star = 0$ case, only trajectories that are exactly identical in terms of strain composition are clustered together. In this case, our clustering amounts to counting mutations that appear on exactly the same strains as one, reducing the number of effective trajectories from 800 to slightly less than 700. For higher values of $d^\star$, the number of clusters steadily goes down until it reaches 1 for $d^\star = 1$, which is the maximum value of the distance $d(X_1, X_2)$. The sharp drop in n for $d^\star = 0.5$ is explained by the high number of very short (typically one time point) and low frequency trajectories that share one out of two strains.

Since the choice of $d^\star$ is arbitrary and since no particular value can be chosen based on the number of clusters $n(d^\star)$, we decide to test our clustering strategy for five values, namely $d^\star \in \{0, 0.05, 0.1, 0.2, 0.49\}$. The bottom panel of figure S1 shows examples of a cluster for the four non-zero values of $d^\star$. The cluster displayed in each case is the one containing the mutation HA1:33R. As $d^\star$ increases, more and more unlike trajectories are grouped together. In the case $d^\star = 0.49$, the cluster consists of 13 trajectories, 5 of which end up dying while the rest fix. Since such a high value of $d^\star$ results in grouping trajectories that do not have the same fate (fixation or death), we decide to exclude it from the rest of the analysis, resulting in four remaining values $d^\star \in \{0, 0.05, 0.1, 0.2\}$.

Once clustering is performed, we re-conduct the analysis of the main text on a set of effective trajectories. This set is constructed by taking one trajectory at random from each cluster. Effective trajectories are then considered independent from each other. The left panel of figure S2 shows the fixation probability of trajectories as a function of their frequency for different values of $d^\star$, for the HA gene of A/H3N2. The result obtained in panel A of figure 2 of the main text is also showed as a reference. For the three lower values of $d^\star$, results do not differ from the one obtained in the main text, even though the number of trajectories in each frequency bin has dropped as can be seen in the right panel of figure S2. This indicates that grouping together trajectories that share most of their strains, and are thus

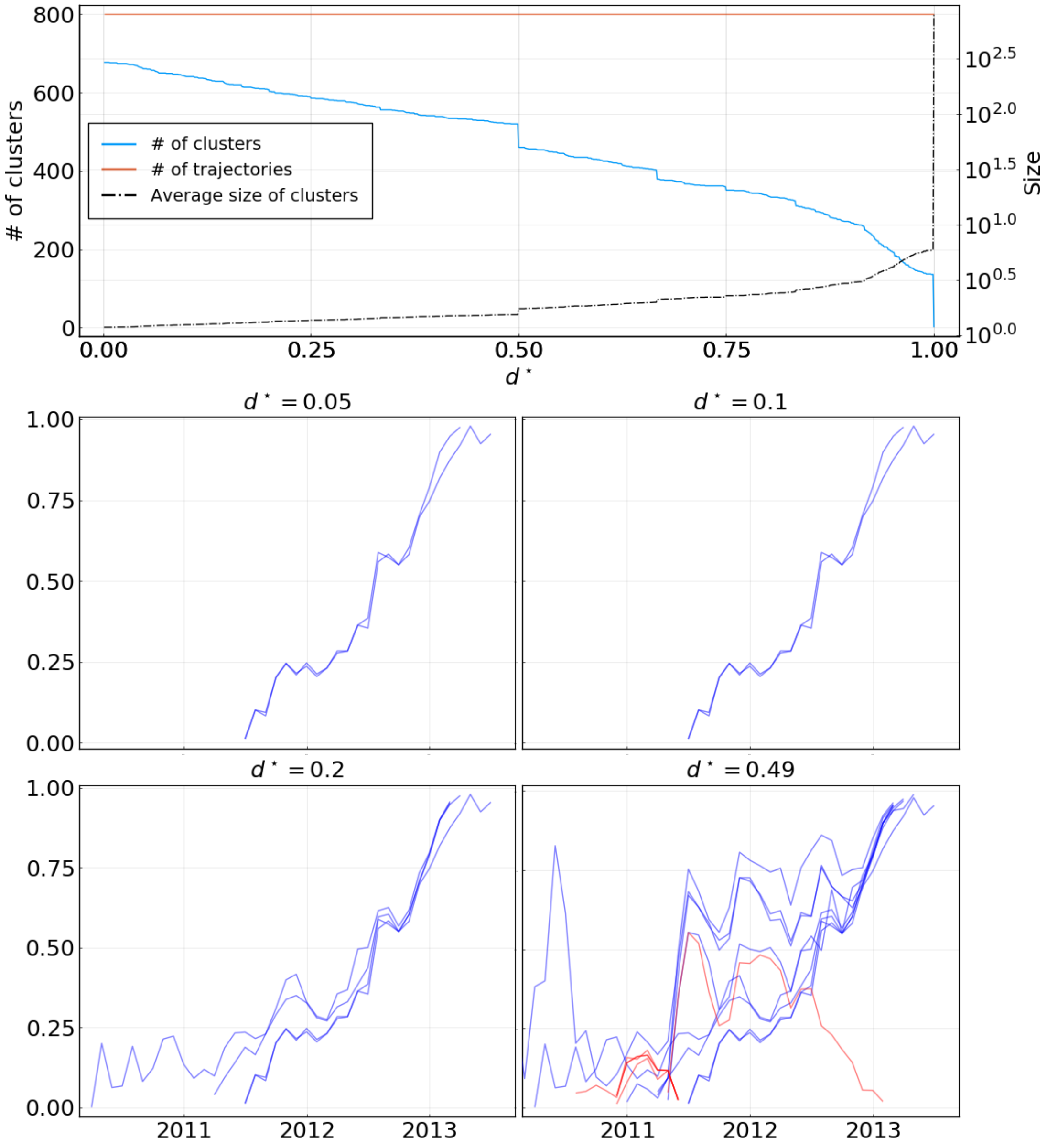

Figure S 1 **Top**: Left-axis: number n of clusters as a function of the threshold distance $d^\star$. The total number of trajectories is shown as a flat orange line. Right-axis: average size of clusters as a function of $d^\star$. **Bottom**: Examples of clusters for four values of $d^\star$. The four clusters displayed are the ones to which the trajectory of mutation HA1:33R belongs.

very correlated, does not modify the computed fixation probability in any way. For the higher value $d^\star = 0.2$, fixation probability drops slightly across all frequency bins, suggesting that fixating trajectories tend to be grouped together more frequently. However, this drop remains of limited amplitude.

Overall, this analysis leads us to think that even though mutations in influenza may be nested, considering trajectories as independent does not result in strong statistical biases. Indeed, clustering similar trajectories together does not significantly modify results presented in the main text.

4. Ability of fitness models to predict fixation

Huddleston et al. (2020) developed several models to predict the future HA population for A/H3N2 influenza. The premise of these models is to assign a fitness score to the HA gene of each A/H3N2 strain in a given year, and to then

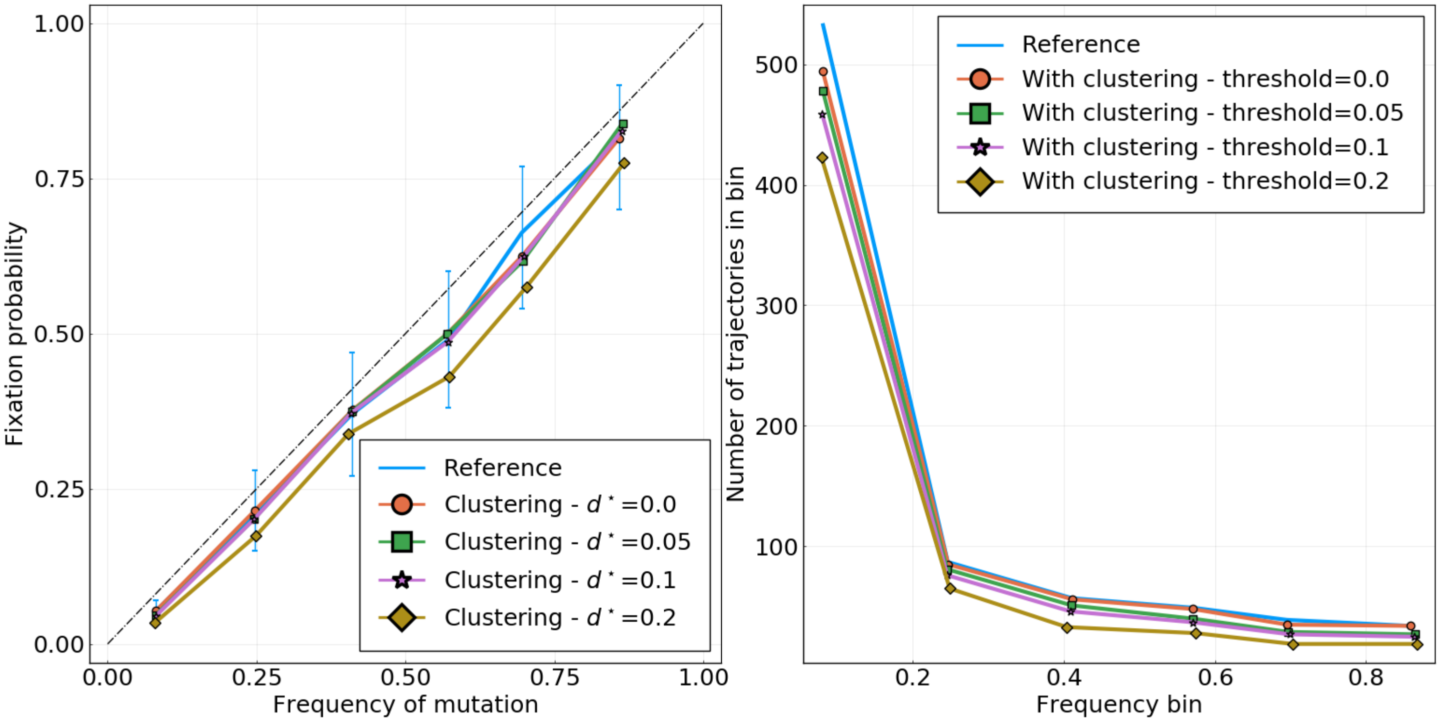

Figure S 2 **Left**: Fixation probability of trajectories as a function of probability for four values of $d^\star$. The reference curve is the same as in panel A of figure 2 for the HA gene. For readability, error bars are only displayed for the reference curve. **Bottom**: Number of trajectories in each frequency bin corresponding to the left panel.

apply deterministic evolutionary equations to obtain the future strain composition. The fitness values are computed using several quantities that have been shown to be connected with viral fitness. Fitness depends either on a single or on two quantities, and coefficient(s) of each fitness model are trained by minimizing the earth movers distance between the observed strain population one year in the future and the estimated population produced by the model. The training is performed using HA sequences from 1990 to 2015, and we consider here the models obtained by taking the average value of coefficients obtained for every year.

We first assess the ability of the four most performing fitness models based on individual scores in (Huddleston et al., 2020) to predict fixation: mutational load (Łuksza and Lässig, 2014), hemagglutination inhibition (HI) antigenic novelty (Neher et al., 2016), a "delta frequency"score based on the recent increase in frequency of clades, and the previously mentioned Local Branching Index (LBI) (Neher et al., 2014). Figure S3 shows fixation probability for HA mutations with fitness scores in the top or bottom half of the fitness distribution for the four fitness measures.

The best performing individual score (and second overall) in (Huddleston et al., 2020), LBI, does not provide any information about fixation (figure S3**A**). This is consistent with the result found in figure 3 of the main text. In panels **B** and **D**, we observe the same inability to predict fixation for the fitness models based on HI titer and delta frequency, which were respectively the second and third best individual scores (fifth and sixth overall) in (Huddleston et al., 2020). However, the score based on mutational load (panel **C**) does show a more significant predicting power, with higher fitness mutations having a $\sim 25\%$ higher chance of fixing than lower fitness ones for three frequency bins in a row. However, this effect vanishes for higher frequencies, with both fitter and less fit mutations having the same chance to fix. This is consistent with the idea that mutations that rose to very high frequency likely have a minor fitness costs even if classified as deleterious mutation.

Next, we use two fitness models based on linear combinations of the following quantities: mutational load + LBI and mutational load + HI antigenic novelty. These composite models were the first and third best performing ones in (Huddleston et al., 2020). Figure S4 again shows fixation probability for HA mutations with fitness scores above or below the median fitness value.

Both models provide some information about fixation, with the mutational load + HI model having more success for frequencies around 0.5. This observation could be explained by the fact that fitness based on HI titers alone showed some power in this same frequency range. However, both models do not seem to perform significantly better than the

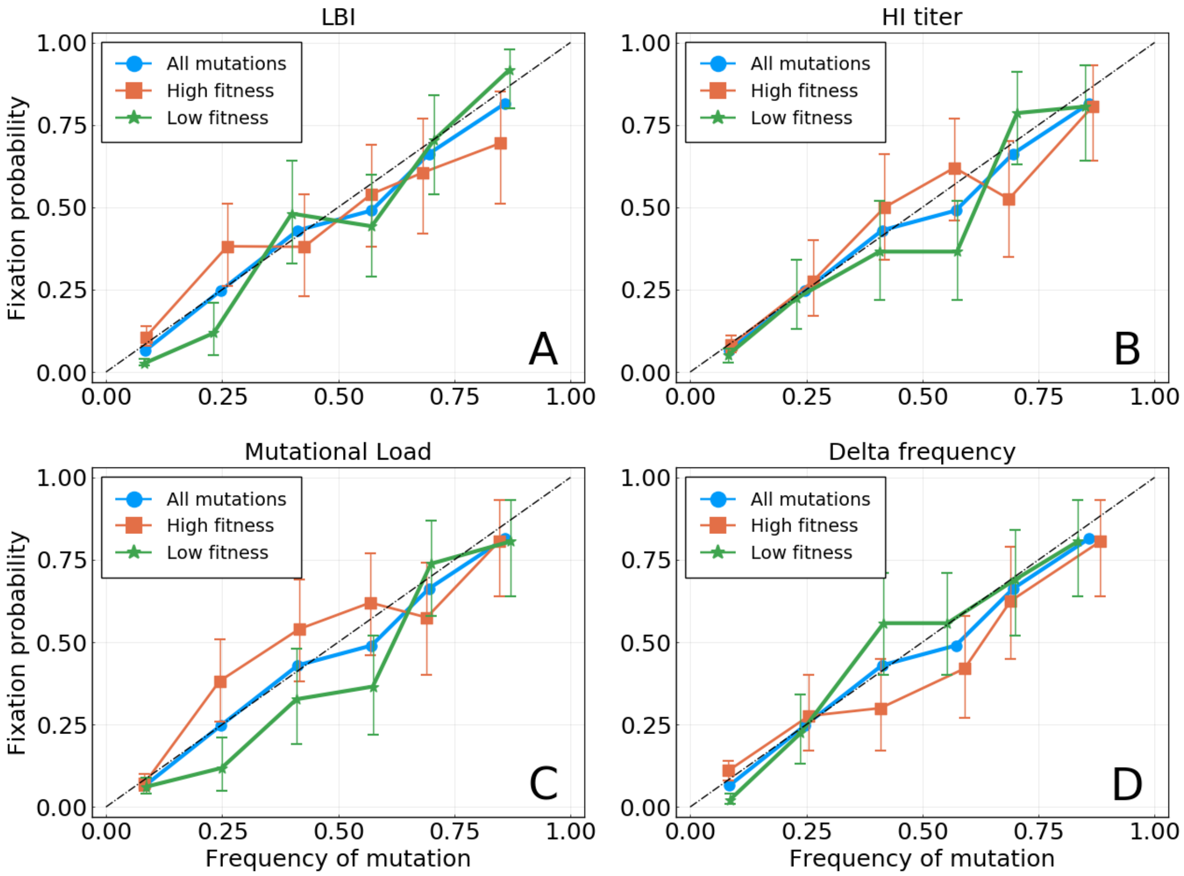

Figure S 3 Ability of fitness scores based on **A** LBI, **B** HI titer, **C** mutational load and **D** delta frequency, to predict fixation of mutation frequency trajectories in the HA gene of A/H3N2. Similar to figure 3 of the main text.

one based on mutational load alone. This is not surprising, as LBI and HI titer did not perform well as individual models.

Combining models in (Huddleston et al., 2020) typically achieved a greater performance than using single models. This was the case for the mutational load + LBI model, combining the second and 7th best predictors overall to obtain the best one, and for the mutational load + HI titer model that combined the 4th and 7th best predictors to obtain the third best one. However, our results indicate that the gain in capability of predicting the future strain composition obtained by combining models is not immediately transferable to the exercise of predicting fixation.

It is important to note that the prediction targets in (Huddleston et al., 2020) and in the present article are not identical. It is shown in figure 4 of the main text that even though LBI performs well when predicting the future strain composition, it may not be because it is indicative of fitness.

5. Biases in frequency estimations

The frequency of mutations in a given time-bin is simply performed by computing their frequency in sequences sampled in that time bin. This leads to potential biases in estimating frequencies, that arise for two reasons:

(i) A mutation present at frequency p in the population might be observed at another frequency $f \neq p$ if f is estimated using a sub-sample of the population.

(ii) For a neutrally evolving population, the distribution of frequencies of alleles is of the form $P(p) \propto 1/p$. This means that the amount of alleles at frequency p is lower when p is higher.

To illustrate *(i)*, let us compute the probability that a mutation present at "real" frequency p in the population is found to be in a given frequency bin $[f_1, f_2]$ when p is estimated from a sample of size n. The sample consists of n

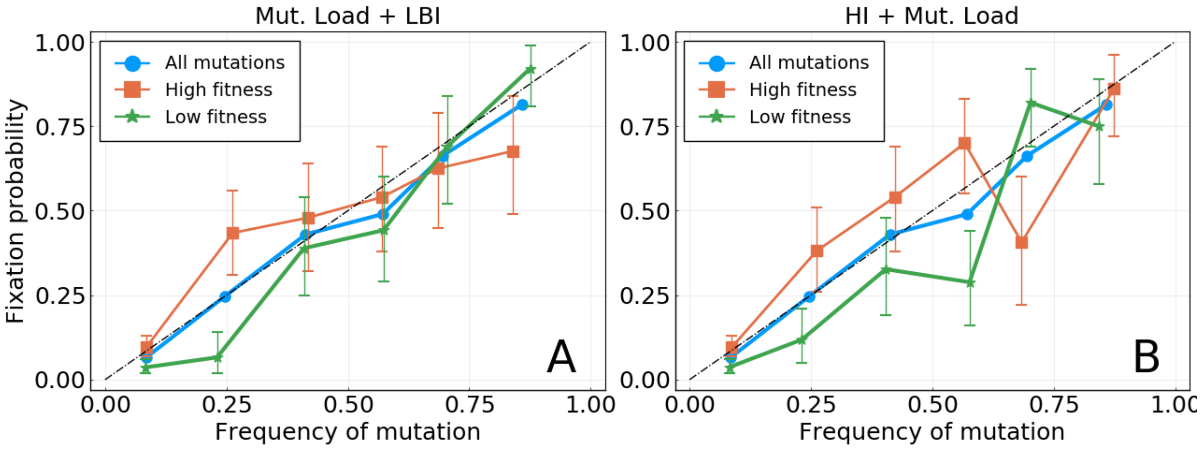

Figure S 4 Ability of fitness scores based on two combinations of the four fitness scores mentioned above: **A** mutational load + LBI; **B** mutational load + HI titer.

observations $\{x_i\}$ with $1 \leq i \leq n$, with $x_i = 1$ if sequence sequence i of the sample bears the mutation, and $x_i = 0$ if not. If n is small with regard to the total population size, we can consider the x_i as random variables with a binomial distribution, meaning that $P(x_i = 1) = p$ and $P(x_i = 0) = 1 - p$. The empirical frequency f is then estimated by taking the average of the x_i variables, that is $f = (x_1 + \ldots + x_n)/n$. If those are independently sampled and n is large enough, the probability of measuring value f is given by the Central Limit Theorem:

$$P_{n,p}(f) \propto e^{(f-p)^2/2\sigma^2}, \;\; \text{where } \sigma^2 = \frac{p(1-p)}{n}. \tag{5}$$

To compute the probability that this mutation is found in a given frequency bin $[f_1, f_2]$, we integrate this distribution:

$$P_{f_1,f_2}(p,n) = \int_{f_1}^{f_2} \mathrm{d}x P_{n,p}(x). \tag{6}$$

Function $P_{f_1,f_2}(p,n)$ is shown as a function of p for a fixed interval and for different values of n in the first panel of figure S5. Note the asymmetry of it: the variance of a binomial distribution of parameter p is small when p is close to 0 or 1, and goes through a maximum at $p = 0.5$. For this reason, mutations present at frequency p close to 0.5 have a higher probability of being observed in other frequency bins. On the contrary, this is unlikely for very rare or very frequent mutations.

We now try to estimate biases in frequency estimation due this phenomenon. Given a set of mutations that have been measured in frequency bin $[f_1, f_2]$, what is the average *real* frequency of these mutations? To compute this, we need to sum $P_{f_1,f_2}(p,n)$ over all possible real frequencies p, giving us the amount of mutations that are observed in interval $[f_1, f_2]$, and weigh this sum by the frequency value p as well as by the background distribution of frequencies $P_b(p) \propto 1/p$. This last quantity represents the expected amount of mutations that are present at frequency p in the population. Note that there is no divergence problem as the smallest non zero frequency is $1/N$, where N is the population size. This leads us to the following expression for the average of "real" frequencies:

$$\begin{aligned} \langle p \rangle (f_1, f_2, n) &= \int_{1/N}^{1-1/N} \mathrm{d}p \, P_{f_1,f_2}(p,n) P_b(p) \, p \\ &= \int_{1/N}^{1-1/N} \mathrm{d}p \, P_{f_1,f_2}(p,n). \end{aligned} \tag{7}$$

We have not made normalization explicit in these equations. It is simply achieved by dividing the above expression by $\int \mathrm{d}p \, P_{f_1,f_2}(p,n) P_b(p)$.

In the second panel of figure S5, $\langle p \rangle (f_1, f_2, n)$ is plotted as a function of the centre of the interval $[f_1, f_2]$ and for different values of n. For sample sizes $n > 100$, the biases due to this effect are almost non existent. For smaller samples, for instance $n = 10$, they are small but non negligible. However, we argue that this is not a significant problem with respect to the main results presented in this article. First, figure S10 shows that sample sizes of the order

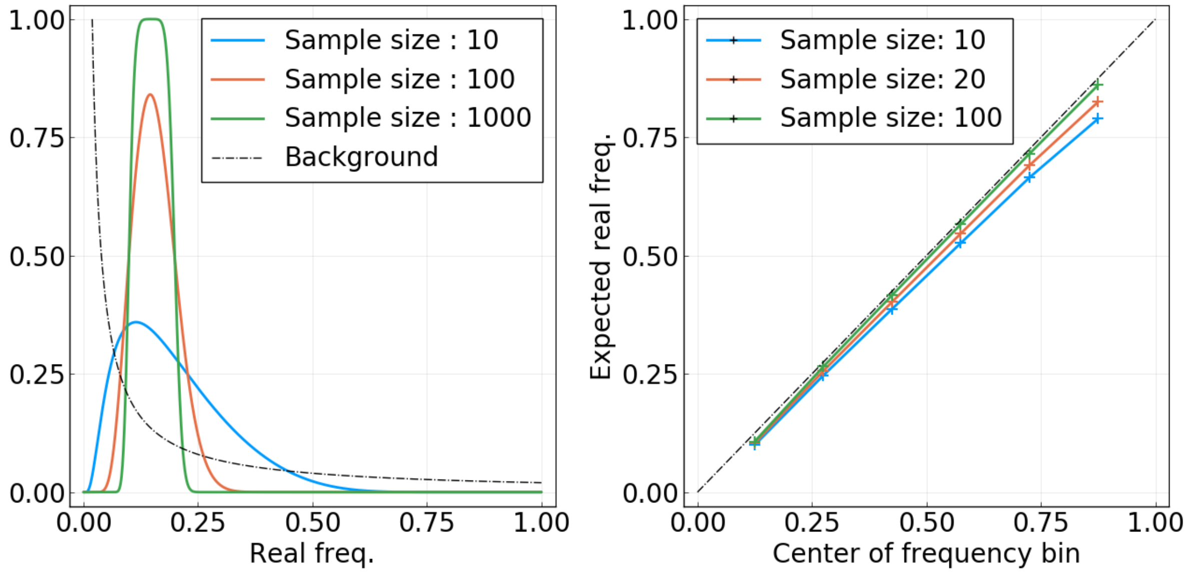

Figure S 5 **Left**: For a mutation present at frequency p in the population, probability of being observed in the frequency bin $[0.1, 0.2]$ as a function of p and for different sample sizes n. The dashed black line sketches the (non-normalized) background distribution $P_b(p)$. **Right**: Expected "real" average frequency of mutations found in frequency bin $[f_1, f_2]$ as a function of the centre of the bin $(f_1 + f_2)/2$, for different sample sizes.

of $n = 10$ are only the case for a few months in the period going from year 2000 to 2018. From 2010 and onwards, more than a hundred sequences are available per month for most months. Secondly, even if most samples were in the $n = 10$ case, deviations shown in figure S5 are small enough that results shown in figures 2 and 3 would be *qualitatively* unchanged.

Note that using the centre of the interval as a reference in figure S5, *i.e.* $(f_1 + f_2)/2$, would be correct in the case of a very large n and a flat background distribution $P_b(p)$. For figures 2 and 3 of the main text however, the average frequency of mutations found in an interval $[f_1, f_2]$ is computed by taking the average of the observed frequencies, and not the centre of the interval. This partially takes into account biases considered here, as the background distribution $P_b(p)$ is then accounted for, even though it is equivalent to assuming infinite sample sizes.

6. Cutting off the HA1 159S branch

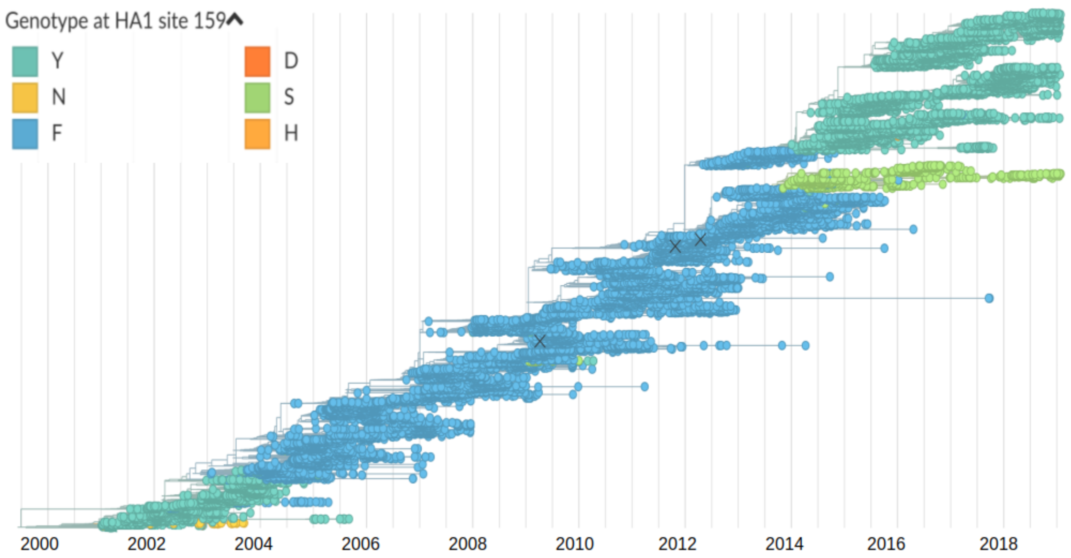

Figure S 6 Tree used for this study, based on a random selection of 100 strains per month from year 2002 to 2018. Nodes and branches are colored according to the amino acid found at position HA1:159. The HA1 159S mutation is visible as a thin but long light-greened color branch, coalescing with the "trunk" around year 2013.

The analysis of the main text is in a large part based on the probability of fixation of mutations. The motivation underlying this choice is the relatively short coalescence time of the A/H3N2 influenza population, typically around three years. This can be seen in figure 2 of the main text, which shows the typical lifetime of frequency trajectories, ending in fixation or loss after at most 3 years in most cases. The tree in figure S6 is another illustration of this: for the most part of it, a "trunk" is clearly identifiable, and lineages that depart from it have a relatively short lifetime. This is no longer the case since the year ~ 2013: two clades have been competing since then, with no definite way to identify a trunk in the tree. The clade defined by the HA1 159S mutation, colored in light green on figure S6, is one of these two competing lineages. Because of this particular situation, the number of mutations fixating in the population is strongly reduced, as a mutation must appear in both clades to reach a frequency of 1. This is a potential flaw in our analysis, which concentrates on mutations fixating.
For this reason, we decided to re-run our analysis after having cut off the HA1 159S clade. In other words, we remove from the set of sequences those that carry the HA1 159S mutation. Results are shown in figures , equivalent to figures 2 and 3 of the main text. It is clear that qualitative results are left unchanged when this competing clade is removed. This can be surprising, as almost no complete fixation of an amino acid mutation has occurred since 2013. Cutting off the HA1 159S branch should thus result in many new fixations, changing the analysis. The reason for the similarity of results can be explained: fixation (resp. loss) of a mutation are defined here as the frequency of this mutation being measured above 95% (resp. 5%) frequency for two months in a row. As the HA1 159S clade is rather sparsely populated, it reaches frequencies lower than 5% two times (in 2015 and 2017), allowing mutations in the competing clade to "fix" as defined here. Thus, removing strains carrying HA1 159S does not introduce a significant amount of "new" fixation events.

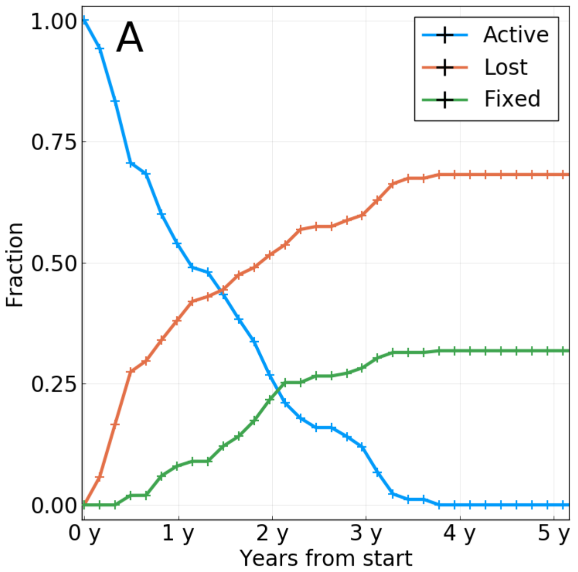

Figure S 7 Equivalent to figure 2 of the main text, but with strains carrying the HA1 159S mutation removed.

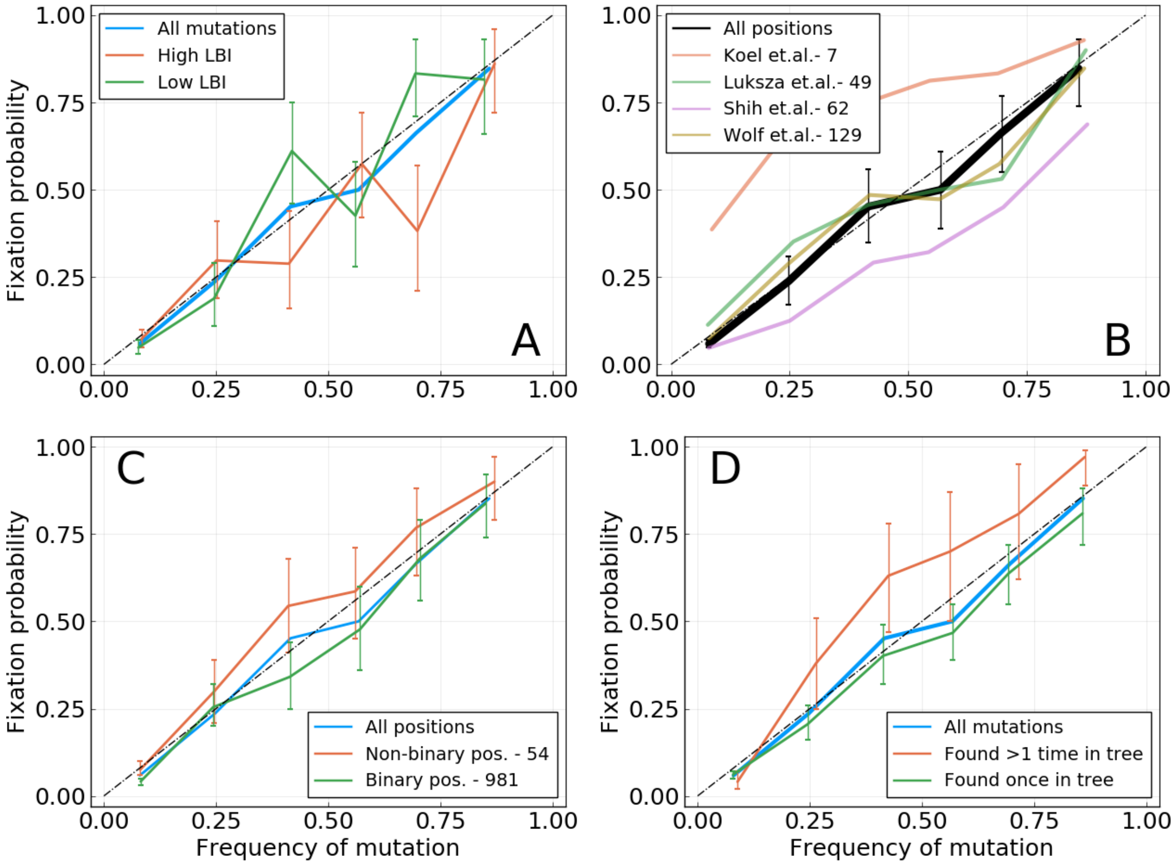

Figure S 8 Equivalent to figure 3 of the main text, but with strains carrying the HA1 159S mutation removed.

7. Probability of fixation in single locus model of evolution

In (Kimura, 1964), Kimura investigates a simple model of evolution with a single locus and a population of size N. In this framework, a mutation at this locus with fitness effect s and observed at frequency f has the following probability of fixation:

$$P_{fix}(f|s, N) = \frac{1 - e^{-sNf}}{1 - e^{-sN}}. \quad (8)$$

Expanding this formula for $sN \ll 1$, that is in the weak selection regime, yields at the first order

$$P_{fix}(f|s, N) = f + f(1 - f)\frac{sN}{2}. \quad (9)$$

Equation 9 tells us two things. First, when the mutation is neutral, that is $s = 0$, we have $P_{fix}(f) = f$. This naturally confirms the result obtained for a neutral model of evolution. Seconds, when $sN \neq 0$, we can expect deviations from the diagonal in a P_{fix} against f plot. The sign of these deviations is determined by the sign of s, with beneficial mutations being found above diagonal while deleterious one are found below. The amplitude of these deviations depends on the strength of selection sN, as well as on the frequency through the $f(1 - f)$ term, making them larger for $f \sim 0.5$.

8. Mutation tables

Gene	Position	AA	Start date	End date	Shih	Luksza	Koel	Tree counts
HA1	144	D	2001-06-09	2002-02-04	true	true	false	0
HA1	189	N	2003-07-29	2004-05-24	false	true	true	2
HA1	159	F	2003-08-28	2004-05-24	false	true	true	2
HA1	226	I	2003-09-27	2004-09-21	true	true	false	3
HA1	145	N	2003-12-26	2004-11-20	false	true	true	2
HA1	227	P	2003-05-30	2005-04-19	false	true	false	2
HA2	32	I	2004-06-23	2005-07-18	false	false	false	1
HA1	193	F	2004-12-20	2006-03-15	false	true	true	1
HA2	46	D	2006-06-13	2007-05-09	false	false	false	2
HA2	121	K	2006-06-13	2007-06-08	false	false	false	1
HA1	50	E	2006-09-11	2007-06-08	false	true	false	2
HA1	140	I	2006-11-10	2007-11-05	true	false	false	1
HA1	173	Q	2007-07-08	2009-01-28	true	true	false	2
HA2	32	R	2007-07-08	2009-01-28	false	false	false	1
HA1	158	N	2009-01-28	2009-07-27	true	true	true	2
HA1	189	K	2009-01-28	2009-07-27	false	true	true	2
HA1	212	A	2009-03-29	2011-01-18	false	false	false	2
HA1	45	N	2010-03-24	2013-02-06	false	false	false	3
HA1	223	I	2010-12-19	2013-02-06	false	false	false	2
HA1	48	I	2011-03-19	2013-02-06	false	false	false	1
HA1	198	S	2011-03-19	2013-02-06	false	false	false	1
HA1	312	S	2009-08-26	2013-03-08	false	false	false	3
HA1	278	K	2011-06-17	2013-03-08	false	true	false	1
HA1	145	S	2011-04-18	2013-04-07	false	true	true	4
HA1	33	R	2011-06-17	2013-06-06	false	false	false	2
HA2	160	N	2012-07-11	2015-09-24	false	false	false	3
HA1	225	D	2013-08-05	2015-09-24	false	false	false	3
HA1	3	I	2013-08-05	2016-11-17	false	false	false	2
HA1	159	Y	2014-02-01	2016-11-17	false	true	true	2
HA1	160	T	2014-01-02	2017-07-15	false	true	false	2

Table S I The 30 trajectories that took place between year 2000 and year 2018 and resulted in fixation. Columns `Shih`, `Luksza` and `Koel` respectively indicate whether the position is found in the epitopes lists in (respectively) (Shih et al., 2007), (Łuksza and Lässig, 2014) and (Koel et al., 2013). The `Tree counts` column indicates the number of times the mutation corresponding to the trajectory can be found in the phylogenetic tree. Note that a trajectory is only shown in the table if the sequenced population counts more than 10 strains at its time of fixation. This explains that only 30 trajectories are displayed, whereas more mutations did fix in this period of time.

Gene	Position	AA	Start date	End date	Fixation	Max. freq.
HA1	106	A	2001-02-09	2002-02-04	lost	1.0
HA1	144	D	2001-06-09	2002-02-04	fixed	1.0
HA1	105	H	2003-04-30	2003-10-27	lost	1.0
HA1	126	D	2003-04-30	2004-05-24	lost	1.0
HA1	140	Q	2004-01-25	2004-06-23	lost	0.31
HA1	226	I	2003-09-27	2004-09-21	fixed	1.0
HA1	173	E	2004-12-20	2006-03-15	lost	0.63
HA1	142	G	2006-06-13	2007-05-09	lost	0.71
HA1	144	D	2006-07-13	2007-05-09	lost	0.67
HA1	128	A	2006-09-11	2007-05-09	lost	0.25
HA1	157	S	2006-09-11	2007-05-09	lost	0.59
HA1	140	I	2006-11-10	2007-11-05	fixed	1.0
HA1	173	N	2007-12-05	2008-07-02	lost	0.3
HA1	157	S	2007-12-05	2008-09-30	lost	0.31
HA1	173	E	2006-06-13	2008-12-29	lost	0.67
HA1	173	Q	2007-07-08	2009-01-28	fixed	0.96
HA1	158	N	2009-01-28	2009-07-27	fixed	0.96
HA1	62	K	2009-01-28	2011-05-18	lost	0.73
HA1	144	K	2009-01-28	2011-05-18	lost	0.75
HA1	62	V	2011-04-18	2011-09-15	lost	0.34
HA1	157	S	2013-05-07	2015-09-24	lost	0.35
HA1	128	A	2012-08-10	2016-11-17	lost	0.81
HA1	197	K	2015-11-23	2016-11-17	lost	0.27
HA1	142	R	2018-05-11	2018-10-08	lost	0.38
HA1	142	G	2012-03-13		poly	0.86
HA1	144	S	2013-12-03		poly	0.96
HA1	121	K	2015-12-23		poly	0.82
HA1	142	K	2016-05-21		poly	0.77
HA1	62	G	2017-03-17		poly	0.75
HA1	128	A	2018-01-11		poly	0.56

Table S II Trajectories of mutations at epitope positions in (Shih et al., 2007) (*Shih et. al.*) that have been observed at least once above frequency 0.25. The `Fixation` column indicates whether the mutation has fixed, disappeared, or is still polymorphic as of October 2018. The `Max.freq.` column indicates the maximum frequency reached by the trajectory. A maximum frequency of 1 for mutations that finally disappear is explained by trajectories reaching frequency 1 for one time bin and going back to lower values for following ones (a frequency above 0.95 for two time bins in a row defines fixation).

Gene	Position	AA	Start date	End date	Fixation	Max. freq.
HA1	50	G	2001-02-09	2002-02-04	lost	1.0
HA1	144	D	2001-06-09	2002-02-04	fixed	1.0
HA1	126	D	2003-04-30	2004-05-24	lost	1.0
HA1	189	N	2003-07-29	2004-05-24	fixed	1.0
HA1	159	F	2003-08-28	2004-05-24	fixed	1.0
HA1	226	I	2003-09-27	2004-09-21	fixed	1.0
HA1	145	N	2003-12-26	2004-11-20	fixed	1.0
HA1	188	N	2004-07-23	2005-02-18	lost	0.36
HA1	227	P	2003-05-30	2005-04-19	fixed	1.0
HA1	173	E	2004-12-20	2006-03-15	lost	0.63
HA1	193	F	2004-12-20	2006-03-15	fixed	0.97
HA1	142	G	2006-06-13	2007-05-09	lost	0.71
HA1	144	D	2006-07-13	2007-05-09	lost	0.67
HA1	157	S	2006-09-11	2007-05-09	lost	0.59
HA1	50	E	2006-09-11	2007-06-08	fixed	0.95
HA1	173	N	2007-12-05	2008-07-02	lost	0.3
HA1	157	S	2007-12-05	2008-09-30	lost	0.31
HA1	173	E	2006-06-13	2008-12-29	lost	0.67
HA1	173	Q	2007-07-08	2009-01-28	fixed	0.96
HA1	158	N	2009-01-28	2009-07-27	fixed	0.96
HA1	189	K	2009-01-28	2009-07-27	fixed	0.96
HA1	213	A	2009-01-28	2010-02-22	lost	0.68
HA1	144	K	2009-01-28	2011-05-18	lost	0.75
HA1	53	N	2009-11-24	2013-02-06	lost	0.72
HA1	278	K	2011-06-17	2013-03-08	fixed	0.98
HA1	145	S	2011-04-18	2013-04-07	fixed	0.99
HA1	159	S	2013-11-03	2015-08-25	lost	0.46
HA1	157	S	2013-05-07	2015-09-24	lost	0.35
HA1	159	Y	2014-02-01	2016-11-17	fixed	0.97
HA1	159	S	2015-10-24	2016-11-17	lost	0.4
HA1	197	K	2015-11-23	2016-11-17	lost	0.27
HA1	160	T	2014-01-02	2017-07-15	fixed	0.96
HA1	142	R	2018-05-11	2018-10-08	lost	0.38
HA1	135	N	2018-06-10	2018-10-08	lost	0.38
HA1	142	G	2012-03-13		poly	0.86
HA1	144	S	2013-12-03		poly	0.96
HA1	121	K	2015-12-23		poly	0.82
HA1	142	K	2016-05-21		poly	0.77
HA1	131	K	2016-09-18		poly	0.77
HA1	135	K	2016-11-17		poly	0.47

Table S III Same as table SII, for (Łuksza and Lässig, 2014) (*Luksza et. al.*).

Gene	Position	AA	Start date	End date	Fixation	Max. freq.
HA1	189	N	2003-07-29	2004-05-24	fixed	1.0
HA1	159	F	2003-08-28	2004-05-24	fixed	1.0
HA1	145	N	2003-12-26	2004-11-20	fixed	1.0
HA1	193	F	2004-12-20	2006-03-15	fixed	0.97
HA1	158	N	2009-01-28	2009-07-27	fixed	0.96
HA1	189	K	2009-01-28	2009-07-27	fixed	0.96
HA1	145	S	2011-04-18	2013-04-07	fixed	0.99
HA1	159	S	2013-11-03	2015-08-25	lost	0.46
HA1	159	Y	2014-02-01	2016-11-17	fixed	0.97
HA1	159	S	2015-10-24	2016-11-17	lost	0.4

Table S IV Same as table SII, for (Koel et al., 2013) (*Koel et. al.*).

9. Supplementary figures

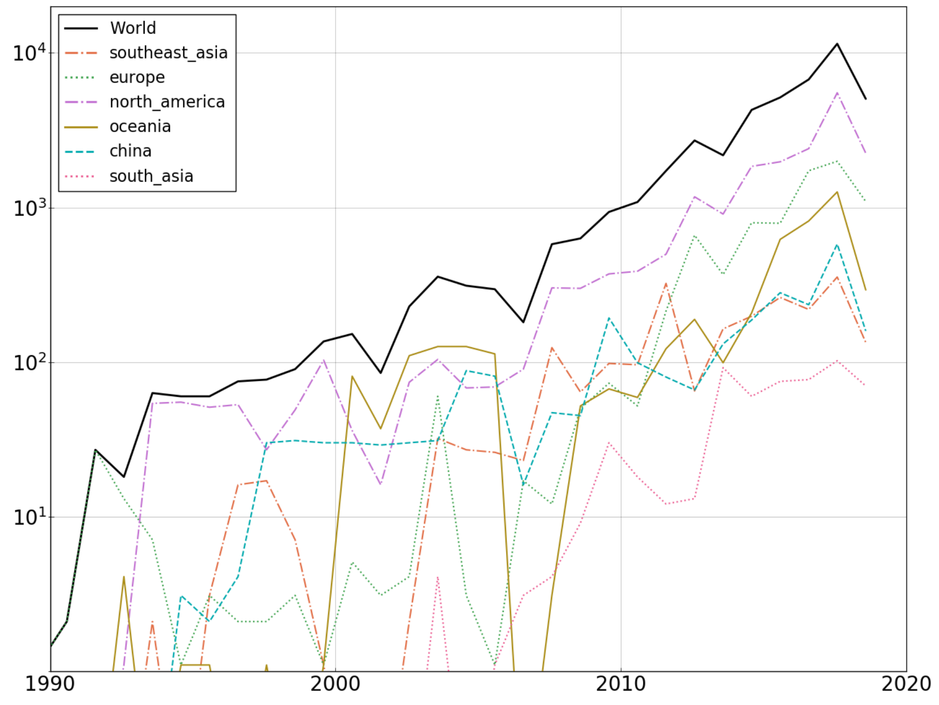

Figure S 9 Number of A/H3N2 HA sequences per year from year 1990.

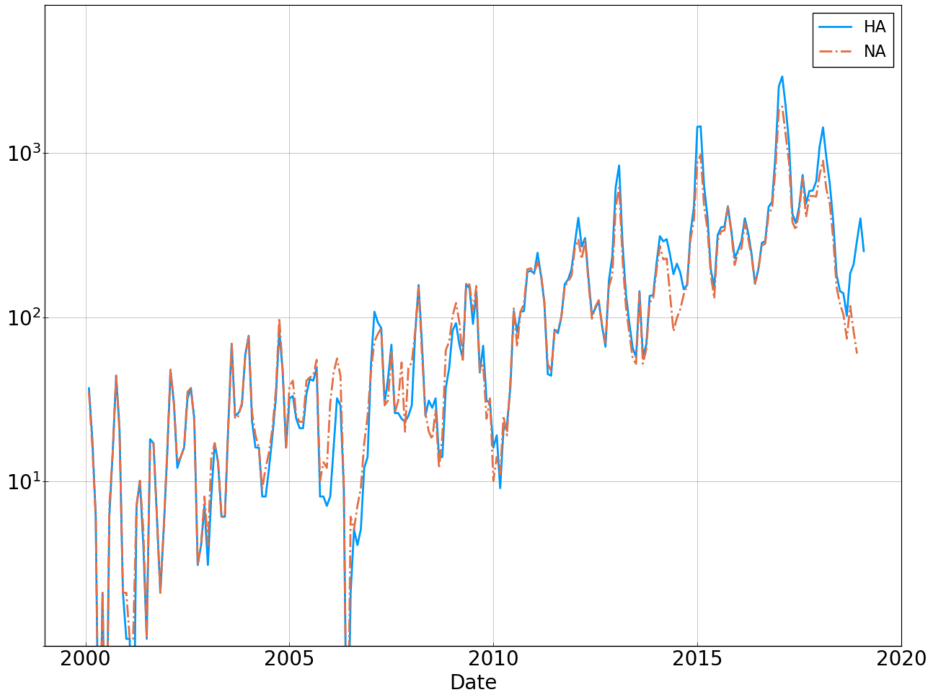

Figure S 10 Number of H3N2 HA and NA sequences per month from year 2000.

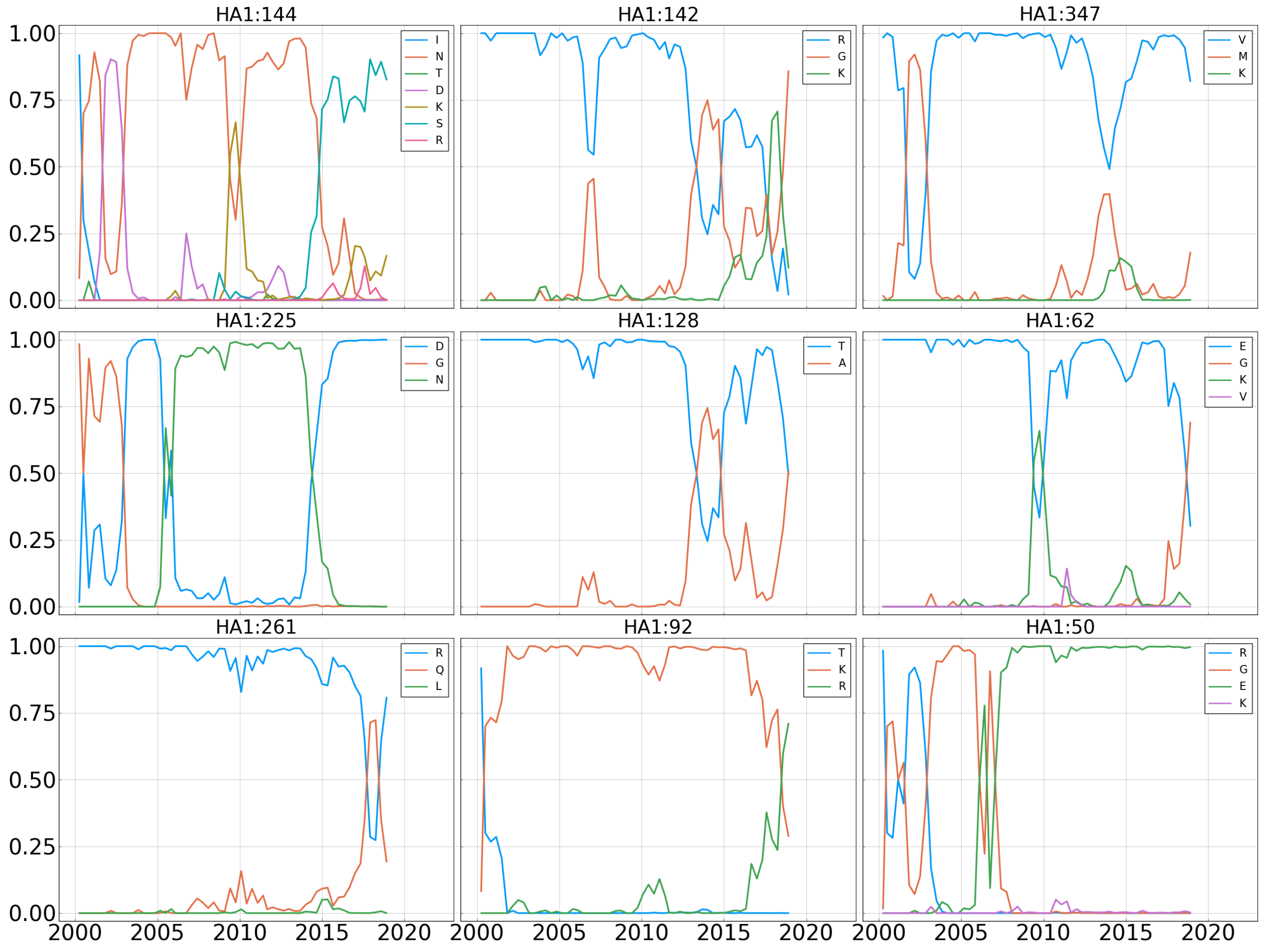

Figure S 11 Frequency trajectories for the 9 most entropic positions in the A/H3N2 HA protein.

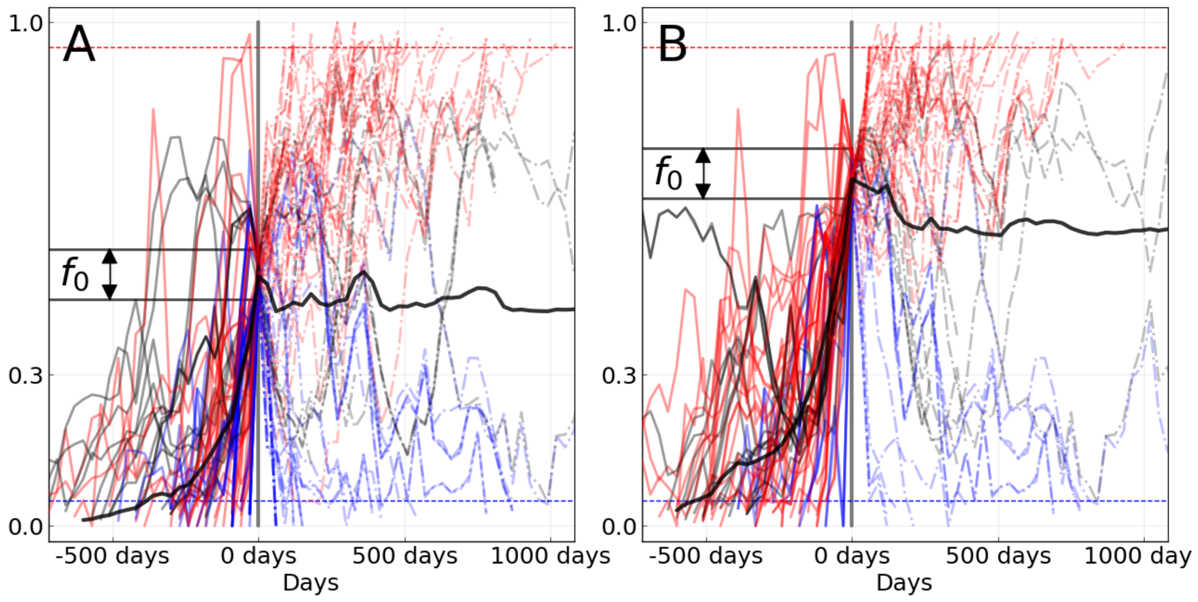

Figure S 12 Equivalent to panel **B** of figure 1 of the main text for A/H3N2, with f_0 equal 0.5 in **A** (76 trajectories), and 0.7 in **B** (63 trajectories).

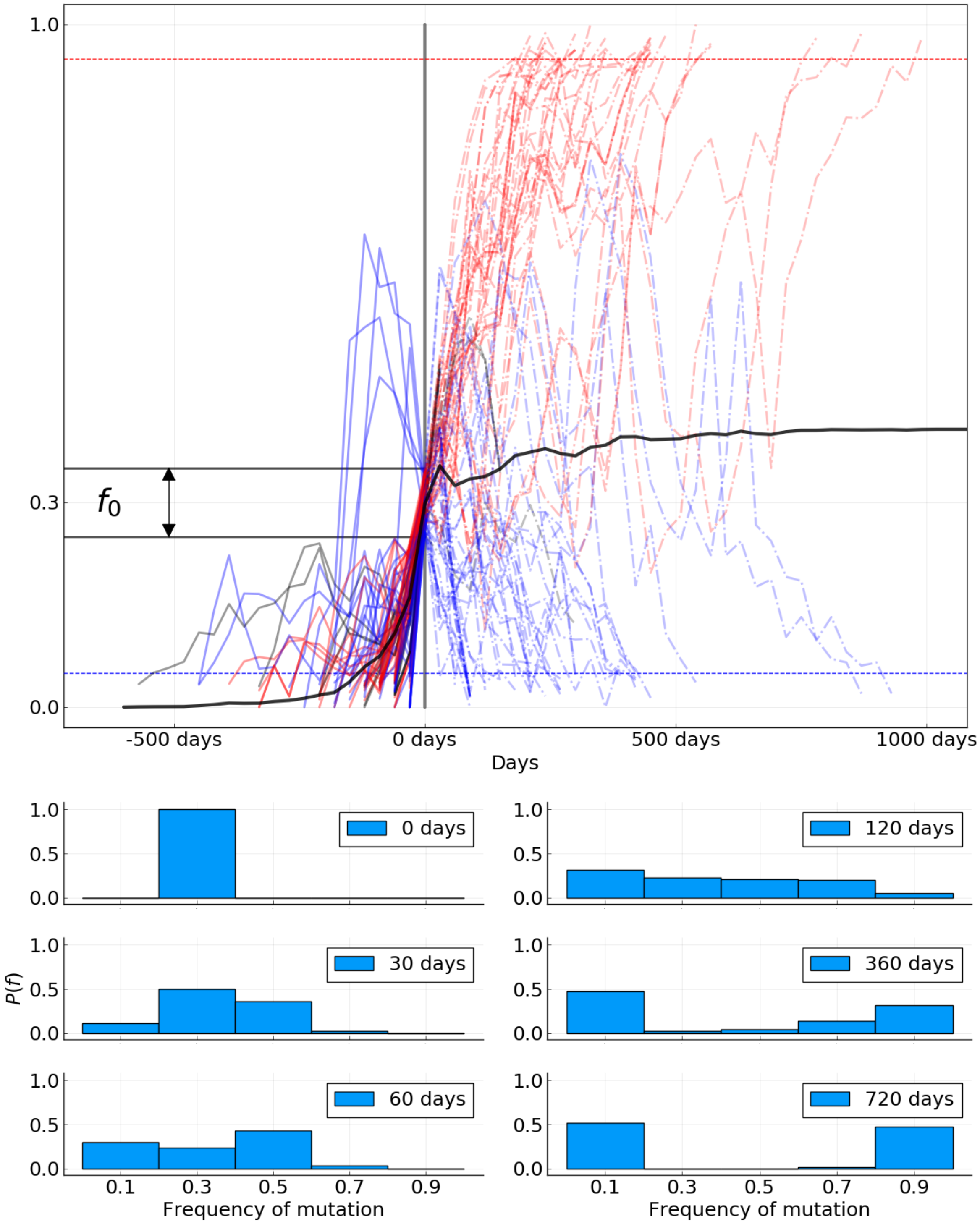

Figure S 13 Equivalent to panels **B** and **C** of figure 1 of the main text for A/H1N1pdm influenza. 89 trajectories are shown and participate to the mean (thick black line).

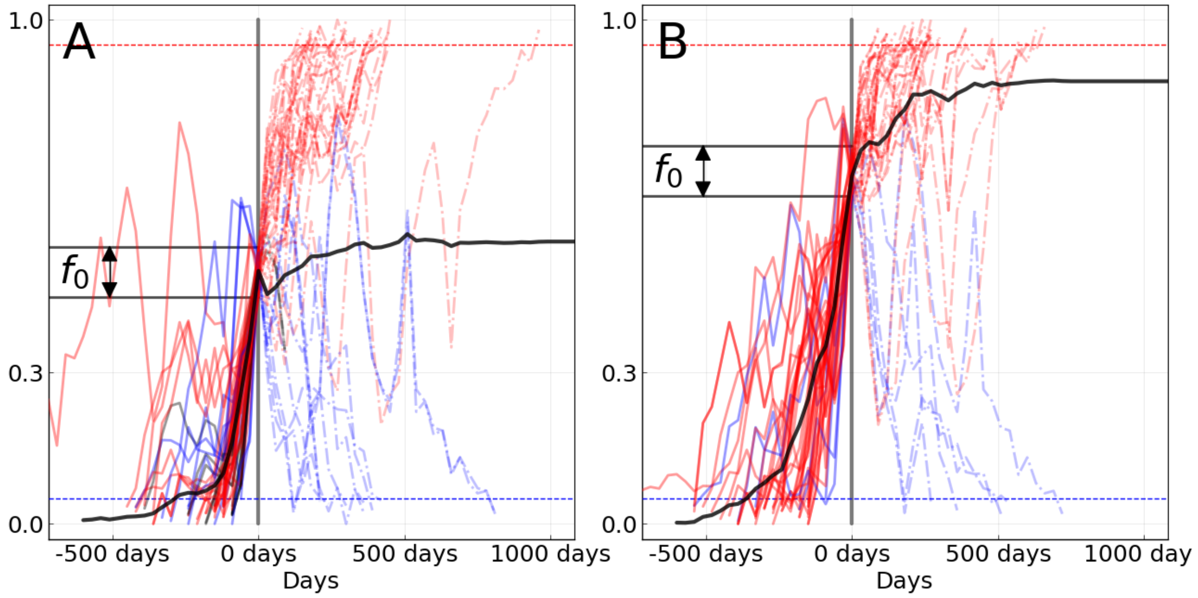

Figure S 14 Equivalent to panel **B** of figure 1 of the main text for A/H1N1pdm, with f_0 equal 0.5 in **A** (50 trajectories), and 0.7 in **B** (41 trajectories).

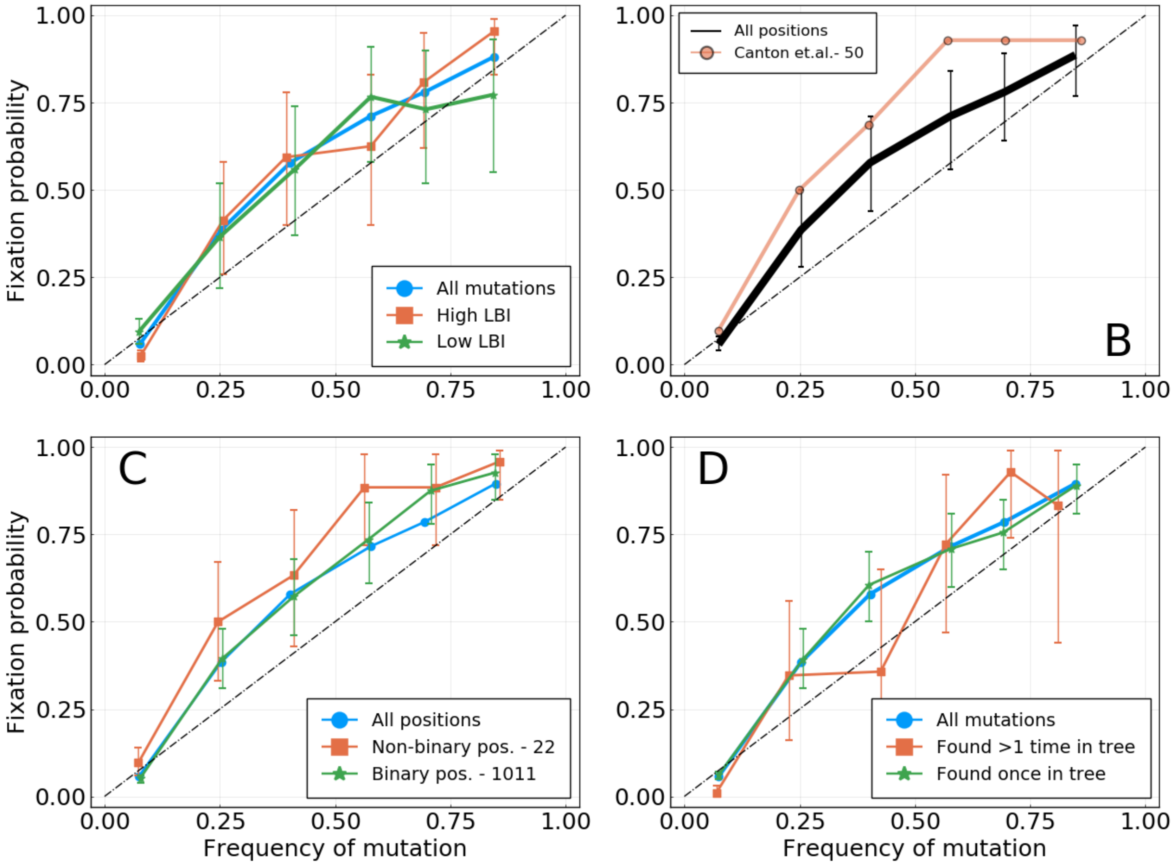

Figure S 15 Equivalent of figure 3 of the main text for the HA gene of A/H1N1pdm influenza. Fixation probability $P_{fix}(f)$ as a function of frequency. **A**: Mutation with higher or lower LBI values, based on their position with respect to the median LBI value. **B**: Different lists of epitope positions in the HA protein. The authors and the number of positions is indicated in the legend. **C**: Mutations for binary positions, *i.e.* positions for which we never see more than two amino acids in the same time bin. **D**: Mutations that appear once or more than once in the tree for a given time bin.

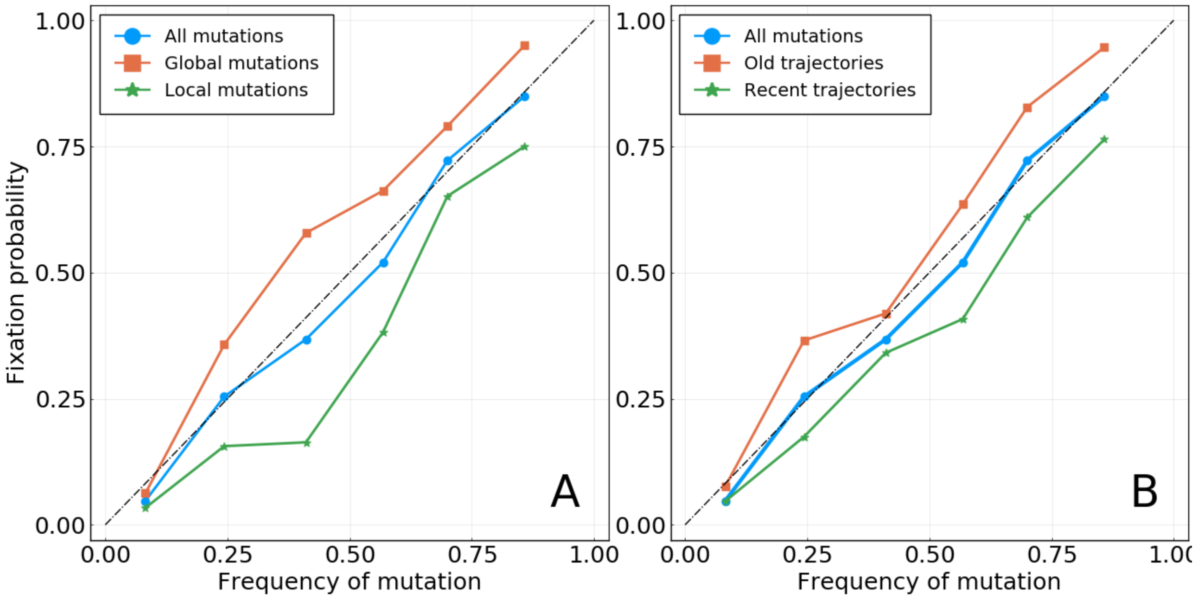

Figure S 16 Based on A/H3N2 HA and NA. **A**: Mutations with a higher or lower geographical spread, based on the median value of the score used (see Methods). *Note*: the words *local* and *global* only reflect the position of the geographic spread of the mutation relative to the median value computed for all mutations found at this frequency. As this median value may change with the considered frequency bin, so does the definition of local and global mutations. **B**: Mutations whose trajectories are older or more recent, based on the median age of trajectories when reaching the considered frequency f.

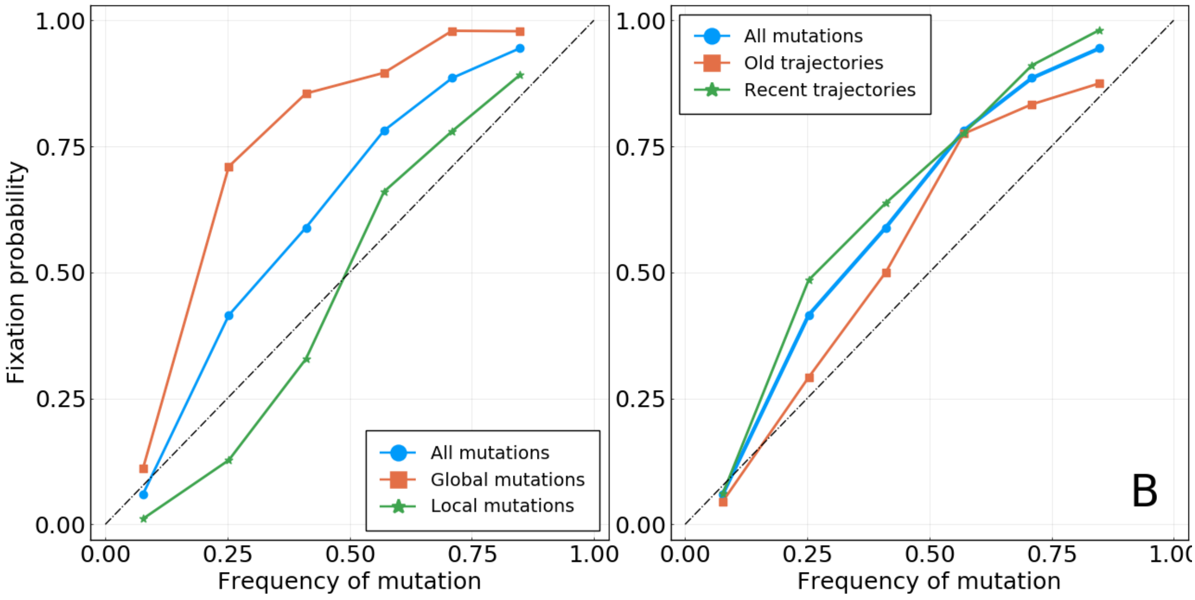

Figure S 17 Based on A/H1N1pdm HA and NA. **A**: Mutations with a higher or lower geographical spread, based on the median value of the score used (see Methods). *Note*: the words *local* and *global* only reflect the position of the geographic spread of the mutation relative to the median value computed for all mutations found at this frequency. As this median value may change with the considered frequency bin, so does the definition of local and global mutations. **B**: Mutations whose trajectories are older or more recent, based on the median age of trajectories when reaching the considered frequency f.

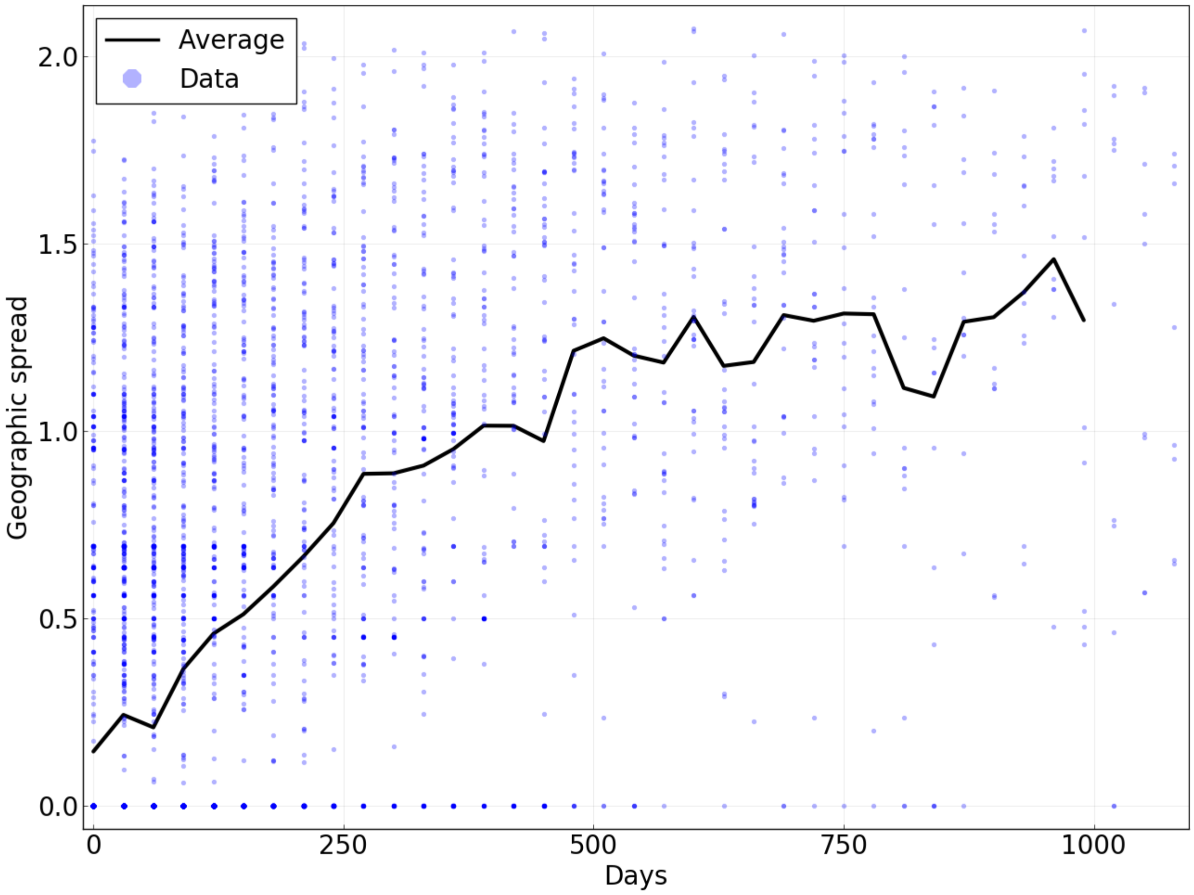

Figure S 18 Geographic spread of mutations as a function of the time for which they have been present in the population above a frequency of 5%. Points represent individual mutations and for a population in a given time bin. The line is the average of dots for a given value on the x-axis. Based on data for A/H3N2 HA.

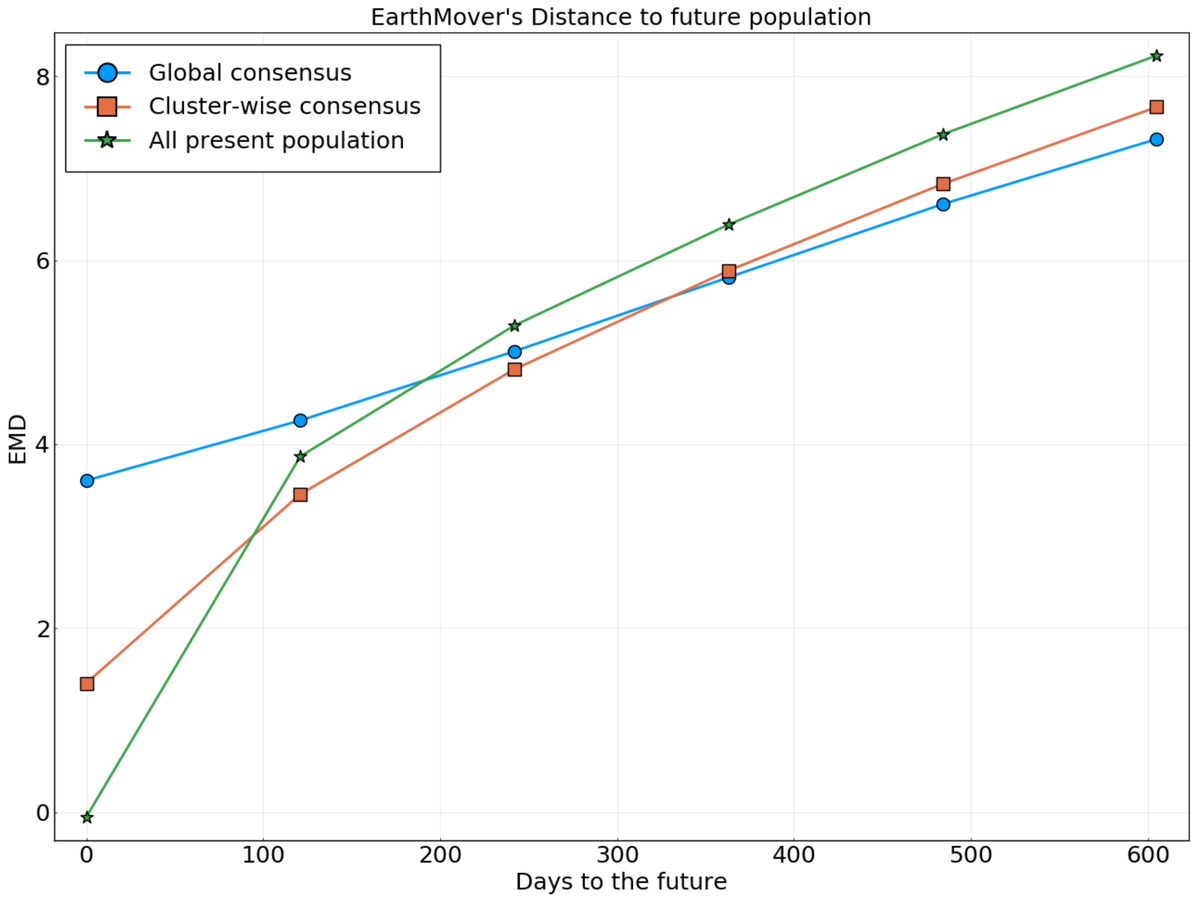

Figure S 19 Earth mover's distance to the future population for different predictors. A present population consists of all A/H3N2 HA sequences sampled in a 4 months time window. Quantities are averaged over all possible "present" populations from the year 2002. Predictors are: **Global consensus**: Consensus sequence of the present population. Best long-term predictor for a structure-less neutrally evolving population. **All present population**: All sequences in the present population. Perfect predictor if the population does not change at all through time. **Cluster-wise consensus**: Consensus sequence for each cluster in the present population. Clusters are based on local maxima of the LBI. Sequences are assigned to a given cluster based on their tree branch-length distance to the corresponding local maximum.

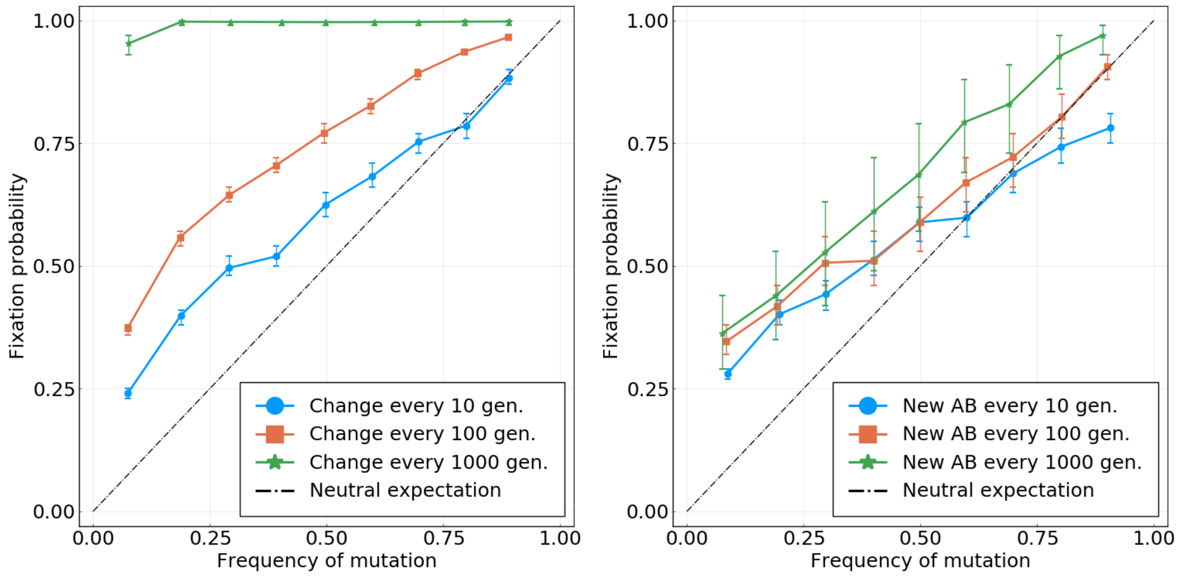

Figure S 20 Fixation probability as a function of frequency for the simulations discussed in the main text. **Left:** Simulation without antibodies. The three colored curves reflect different rate of change for the fitness landscape. Visual inspection of the frequency trajectories indicates a typical sweep time of ~ 400 generations. **Right:** Simulation with antibodies. The different colored curves indicate the rate at which antibodies are introduced.

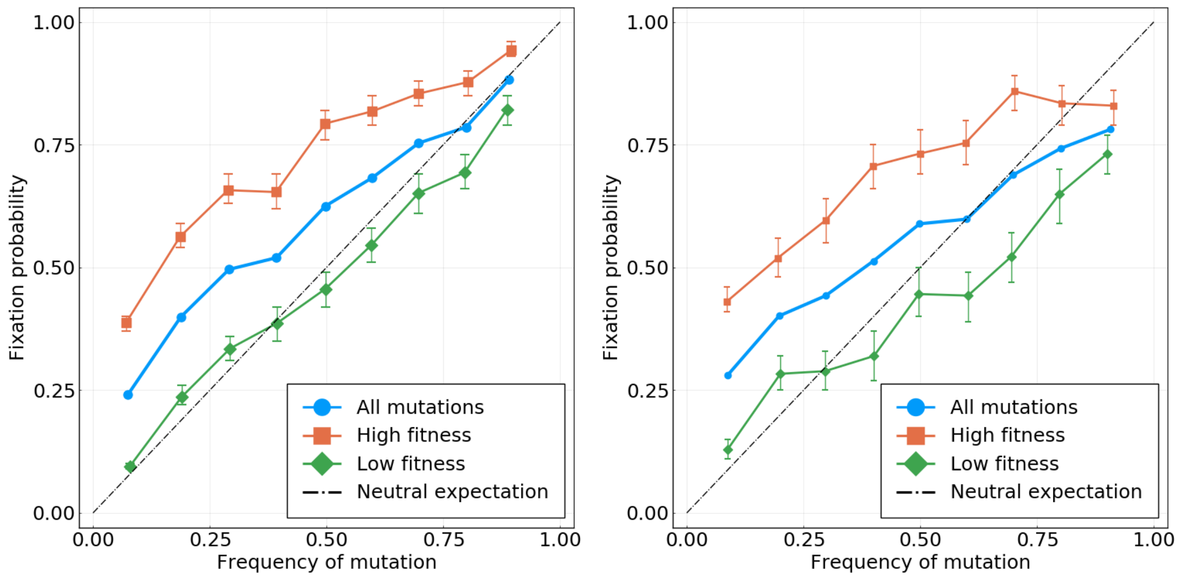

Figure S 21 Fixation probability as a function of frequency for the simulations discussed in the main text, with trajectories stratified according to real fitness values. "High" and "low" fitness classes are defined with respect to the median value. **Left:** Simulation with a purely additive landscape that changes every $\Delta t = 10$ generations. **Right:** Simulation with antibodies, with a new antibody every $\Delta t = 10$ generations.
